# Recent Advances in Decoupling Strategies for Soft Sensors

**DOI:** 10.1002/advs.202514499

**Published:** 2026-02-09

**Authors:** Yangbo Yuan, Wanqing Zhang, Jia‐Yu Yang, Manqi Peng, Huanyu Cheng

**Affiliations:** ^1^ Department of Engineering Science and Mechanics The Pennsylvania State University University Park Pennsylvania USA; ^2^ Institute of Medical Science and Technology National Sun Yat‐sen University College of Medicine Kaohsiung Taiwan; ^3^ Weiyang College Tsinghua University Beijing China

**Keywords:** decoupling strategies, insensitive, machine learning, soft sensors

## Abstract

Soft sensors have shown great promise in emerging fields such as wearable electronics, soft robotics, personalized healthcare, and human‐machine interaction. However, their practical deployment remains limited due to signal interference and cross‐sensitivity arising from simultaneous mechanical and environmental stimuli. To address these challenges, this review presents an overview of recent signal decoupling strategies for accurate and stable sensing. First, approaches for suppressing interference from individual physical parameters, such as stretching, bending, temperature, humidity, pressure, and light, are discussed. Next, array‐level crosstalk elimination techniques are reviewed to ensure reliable spatial resolution. Furthermore, advanced strategies based on spatiotemporal separation and machine learning are summarized for decoupling complex and coupled input signals. These approaches offer promising routes to simplify sensor design, reduce system complexity, and enhance signal fidelity. Finally, remaining challenges and future directions are discussed to guide the development of high‐performance, scalable, and integrable sensing systems.

## Graphical abstract

This review provides an overview of recent advances in decoupling strategies for soft sensors. It summarizes single‐modal sensors that are insensitive to stretching, bending, crosstalk, and other environmental interferences, and highlights emerging multimodal decoupling methods enabled by spatiotemporal information and machine learning. Key challenges of machine learning‐based strategies are discussed, along with potential solutions for improving robustness and generalization.

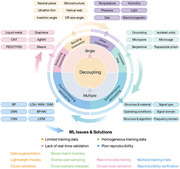

## 1 Introduction

**FIGURE 1:**
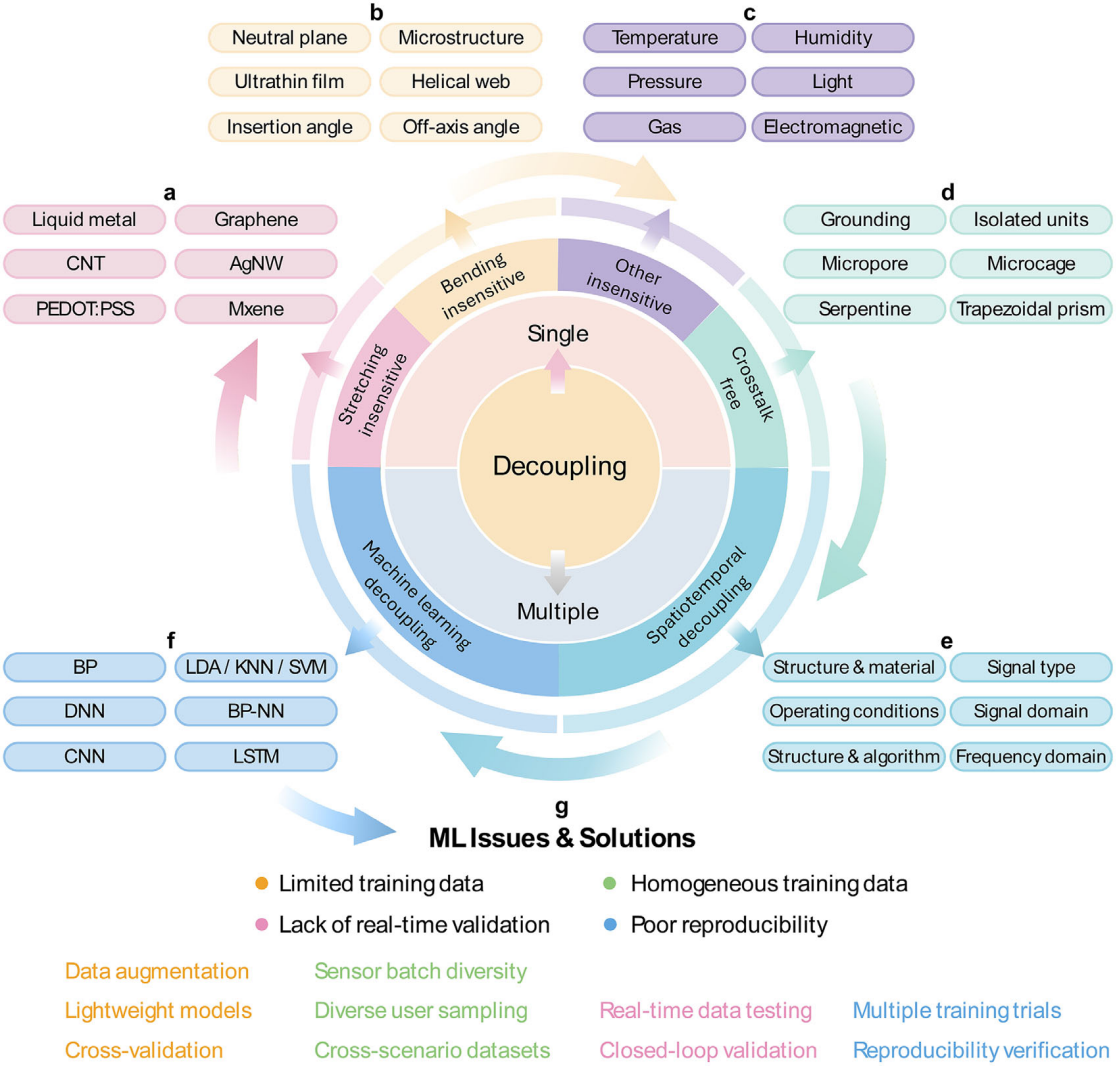
Overview of decoupling strategies in soft sensors. (a) Stretching‐insensitive strategies based on various functional materials. (b) Bending‐insensitive strategies achieved through structural engineering. (c) Decoupling strategies for environmental interferences, including temperature, humidity, pressure, light, gas, and electromagnetic. (d) Crosstalk‐free strategies for sensor arrays. (e) Spatiotemporal strategies for decoupling multiple physical inputs. (f) Machine learning (ML)‐assisted decoupling strategies for complex multimodal signals. (g) Key challenges and corresponding solutions in ML‐based decoupling applications.

To emulate the complex and refined sensing functions of human skin, flexible electronics have advanced not only in single‐function devices but also in multimodal systems capable of simultaneously detecting multiple physical stimuli [1, 2, 3, 4, 5]. The development of such sensors relies primarily on two factors: 1) functional materials that respond selectively to various inputs, and 2) structural designs that deform differently under distinct mechanical stimuli. Together, these features enable concurrent sensing of varying mechanical and environmental cues for applications in robotics [6, 7, 8], human‐machine interfaces [9, 10, 11], and health monitoring [12, 13, 14, 15]. However, the presence of multiple concurrent stimuli often leads to signal interference due to cross‐sensitivity, compromising sensing accuracy. Such interference can arise not only from mechanical coupling between stretching and bending but also from environmental factors such as temperature fluctuation, humidity variation, and ambient pressure, which cause unstable or overlapping output signals. To address this challenge, efforts have been devoted to improving selectivity and stability via different decoupling strategies. Building upon these advances, this review summarizes the latest developments in signal decoupling strategies for soft sensors (Figure 1).

The discussion begins with single‐parameter decoupling strategies. For instance, stretching‐insensitive designs can utilize materials such as liquid metals (LM) [23], graphene [24, 25], carbon nanotubes (CNTs) [26], silver nanowires (AgNWs) [27, 28], Poly(3,4‐ethylenedioxythiophene):poly(styrene sulfonate) (PEDOT:PSS) [29], and MXene [30, 31]. Next, bending‐insensitive approaches are introduced to feature structural innovations, including neutral mechanical plane [43], microstructured geometries [44], ultrathin conductive films [45, 46, 47], helical web designs [48], optimized installation angles [49], and off‐axis configurations [50]. Environmental interferences, such as those caused by temperature [51, 52, 58, 89], humidity [53, 54, 59], gas [55, 56], pressure [60], and light [57, 61], can be addressed through targeted suppression techniques. To improve spatial resolution, crosstalk‐free strategies in sensor arrays are also reviewed, including approaches based on common grounded electrodes [72], isolated sensing units [73], micropore isolation layer [74], microcage structures [75], serpentine interconnects [76], and trapezoidal prism designs [44].

Furthermore, the article reviews spatiotemporal strategies for decoupling multimodal signals [77, 78, 79, 80, 81, 82, 83, 84, 85, 86, 87, 88, 89, 90, 91, 92, 93, 94, 95], and 3D forces [96, 97, 98, 99, 100, 101, 102, 103, 104, 113]. In addition, various machine learning (ML) models are discussed for their effectiveness in recognizing and separating complex, coupled signals [109, 110, 111, 112, 113, 114]. Finally, key implementation challenges of ML‐based decoupling, such as limited and homogeneous training data [116, 117, 118, 122], lack of real‐time validation [125], and poor reproducibility [127], are analyzed, with potential solutions proposed to enhance robustness and practical applicability [119, 120, 121, 123, 124, 126, 128].

## 2 Fundamentals of Soft Sensors

### 2.1 Categories of Soft Sensors

**FIGURE 2:**
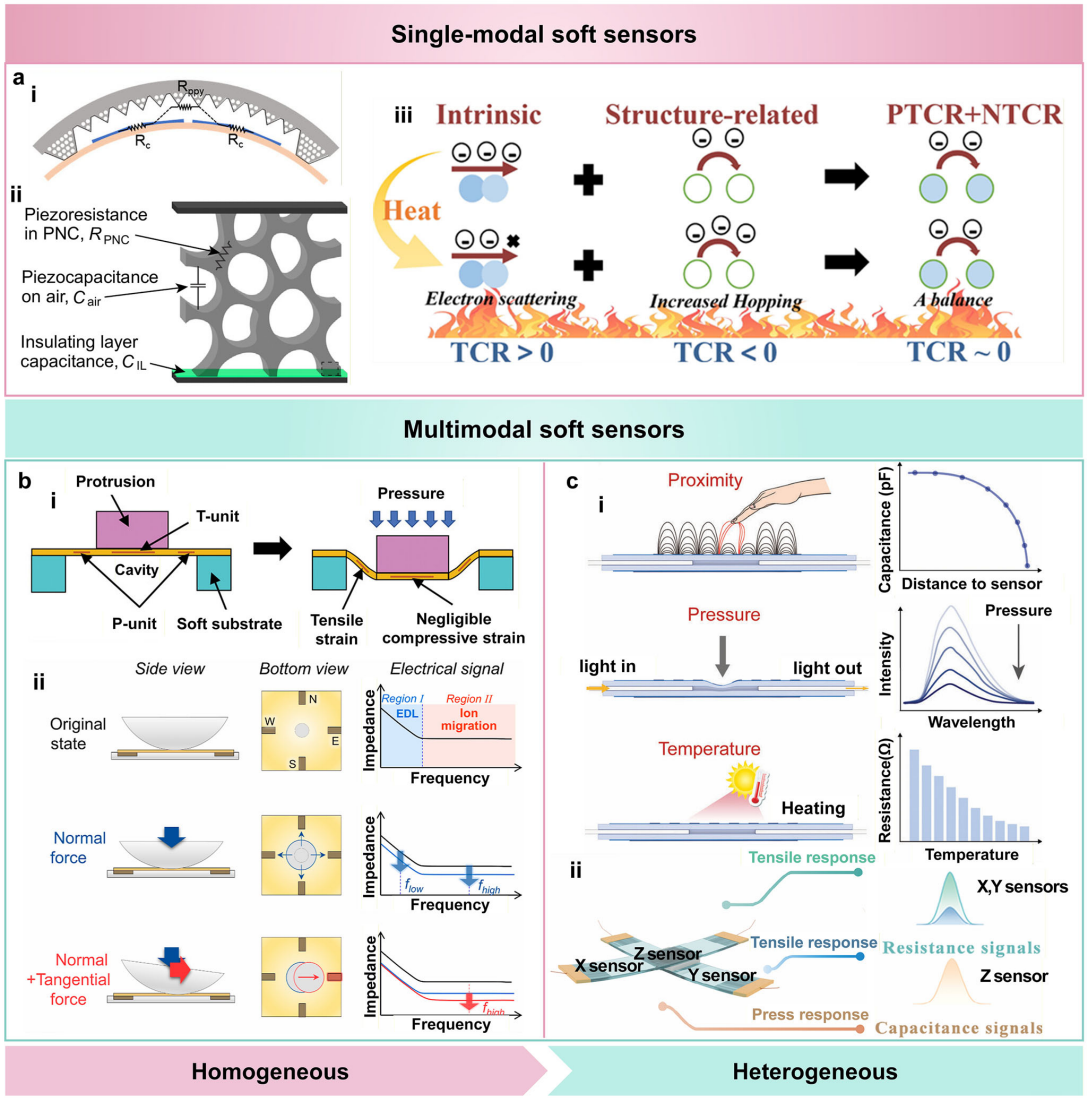
Classification of soft sensors. (a) Single‐modal soft sensors designed to detect a specific stimulus. (i) Working principle of a typical piezoresistive soft sensor, where external pressure alters the conductive pathways and contact resistance. Reproduced with permission [44]. Copyright 2022, Springer Nature. (ii) Piezoresistive–capacitive hybrid mechanism in porous nanocomposite (PNC), in which the overall response arises from both resistance (R_PNC_) and capacitance (C_air_ and C_IL_) in conductive domains. Reproduced with permission [26]. Copyright 2024, Elsevier. (iii) Temperature‐insensitive mechanism achieved by balancing positive and negative temperature coefficient resistances (PTCR + NTCR). Reproduced with permission [58]. Copyright 2023, Elsevier. (b) Homogeneous multimodal soft sensors that distinguish multiple stimuli within a single material system. (i) Pressure–temperature decoupling via cavity‐protrusion design that separates deformation‐sensitive (P‐unit) and temperature‐sensitive (T‐unit) regions. Reproduced with permission [93]. Copyright 2023, Wiley‐VCH. (ii) Frequency‐domain analysis enabling the differentiation of normal and tangential forces based on impedance spectra. Reproduced with permission [104]. Copyright 2023, American Chemical Society. (c) Heterogeneous multimodal sensor integrating distinct transduction mechanisms for multifunctional sensing. (i) Independent proximity, pressure, and temperature sensing enabled by capacitive, optical, and resistive domains, respectively. Reproduced with permission [95]. Copyright 2023, Wiley‐VCH. (ii) Decoupled X/Y/Z‐axis responses showing resistive and capacitive signal differentiation. Reproduced with permission [94]. Copyright 2024, Wiley‐VCH.

Soft sensors can be broadly categorized into single‐modal and multimodal types. Single‐modal sensors are designed to detect a specific physical or chemical stimulus, with significant efforts devoted to enhancing their performance parameters such as sensitivity, stability, and mechanical compliance under single‐mode operation (Figure 2a) [26, 44, 58]. Soft multimodal sensors refer to deformable sensing systems that integrate multiple signal transduction mechanisms within flexible or stretchable substrates to simultaneously detect diverse physical or chemical stimuli. According to their structural configuration, soft multimodal sensors can be further divided into homogeneous and heterogeneous types. Homogeneous sensors employ similar materials and the same transduction mechanism to differentiate multiple stimuli by leveraging material anisotropy or structural design (Figure 2b) [93, 104], whereas heterogeneous sensors integrate multiple sensing elements based on distinct physical or chemical principles, enabling the simultaneous detection of different signals (Figure 2c) [94, 95]. Moreover, array configurations can be incorporated into both types to achieve spatially resolved and crosstalk‐free sensing.

### 2.2 Sensing Mechanisms of Soft Sensors

**FIGURE 3:**
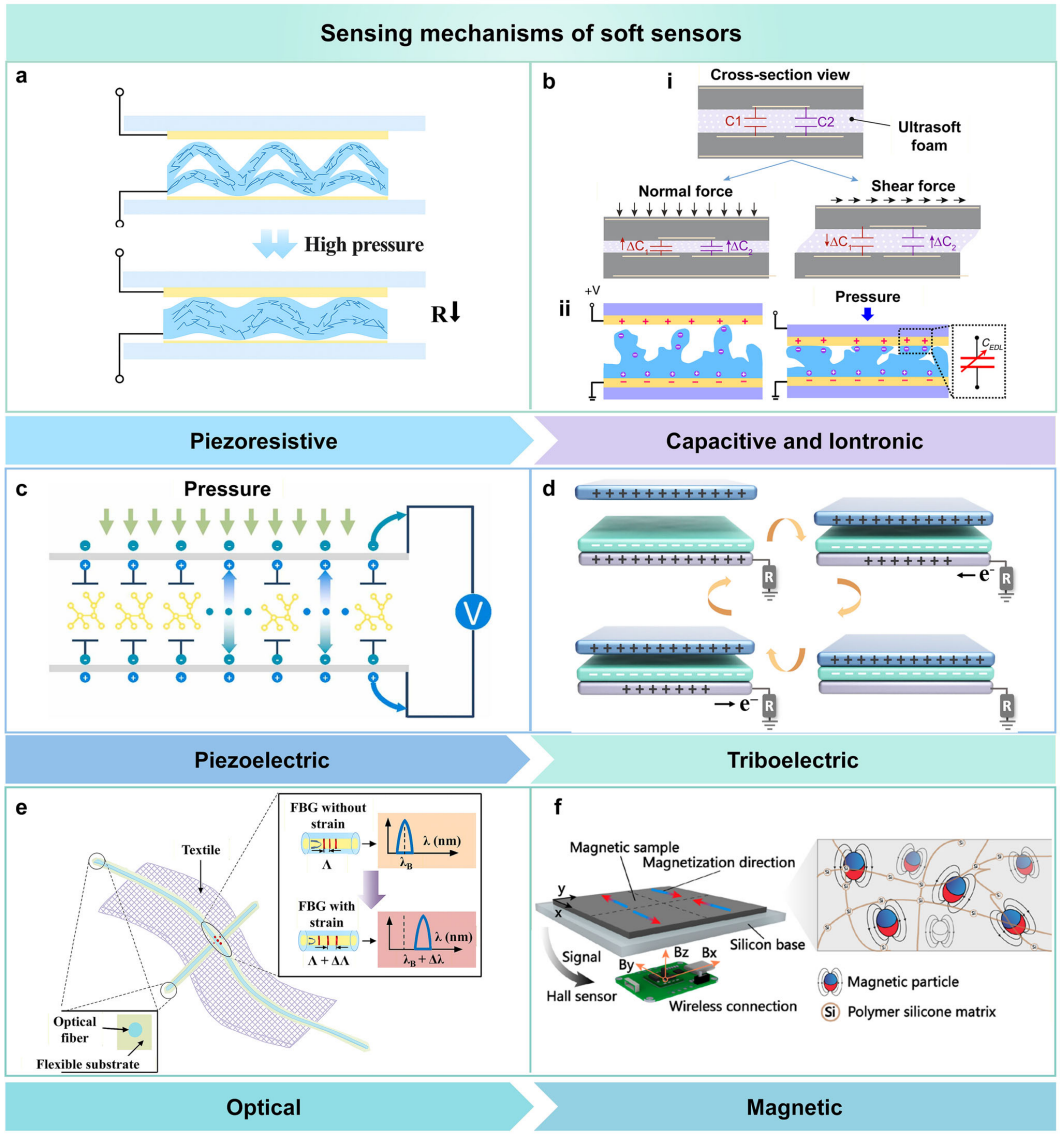
Representative sensing mechanisms in soft sensors. (a) Piezoresistive sensing mechanism based on resistance variation under applied pressure. Reproduced with permission [16]. Copyright 2025, Wiley‐VCH. (b) Capacitive and iontronic sensing mechanisms. (i) Dual‐capacitor layout for distinguishing normal and shear forces. Reproduced with permission [17]. Copyright 2022, Springer Nature. (ii) Ionic transport under pressure enabling iontronic signal generation. Reproduced with permission [18]. Copyright 2022, Springer Nature. (c) Piezoelectric sensing via dipole reorientation under pressure to generate an electric signal. Reproduced with permission [19]. Copyright 2022, Elsevier. (d) Triboelectric mechanism involving charge transfer between contact surfaces. Reproduced with permission [20]. Copyright 2022, American Association for the Advancement of Science. (e) Optical fiber‐based sensing via wavelength shift in fiber Bragg gratings (FBGs) embedded in textiles under strain. Reproduced with permission [21]. Copyright 2022, IEEE. (f) Magnetic sensing using embedded magnetic particles and Hall sensors to monitor magnetic flux changes caused by mechanical deformation. Reproduced with permission [22]. Copyright 2022, American Chemical Society.

The sensing mechanisms of soft sensors describe the process that converts external physical or chemical stimuli into measurable electrical signals through the deformation or property variation of functional materials. Depending on the type of energy or signal transduction, the most common mechanisms include piezoresistive [16], capacitive [17], iontronic [18], piezoelectric [19], triboelectric [20], optical [21], and magnetic effects (Figure 3) [22]. Each mechanism offers unique advantages in sensitivity, response speed, and adaptability, yet the coexistence of multiple transduction pathways in sensing systems often leads to signal coupling. Therefore, a thorough understanding of these fundamental mechanisms forms the basis for designing effective signal acquisition and decoupling strategies in soft sensors.

### 2.3 Signal Acquisition Methods for Soft Sensors

In order to obtain clear and reliable signals for subsequent decoupling, signal acquisition plays a crucial role in sensing systems. The quality of the acquired signals directly determines the complexity and accuracy of the following decoupling process. In recent years, significant progress has been made in signal acquisition for soft sensors, evolving from direct electrical readout of raw signals to integrated multi‐channel acquisition circuits, frequency‐domain readout, and finally to artificial intelligence (AI)‐assisted adaptive signal processing.

**FIGURE 4:**
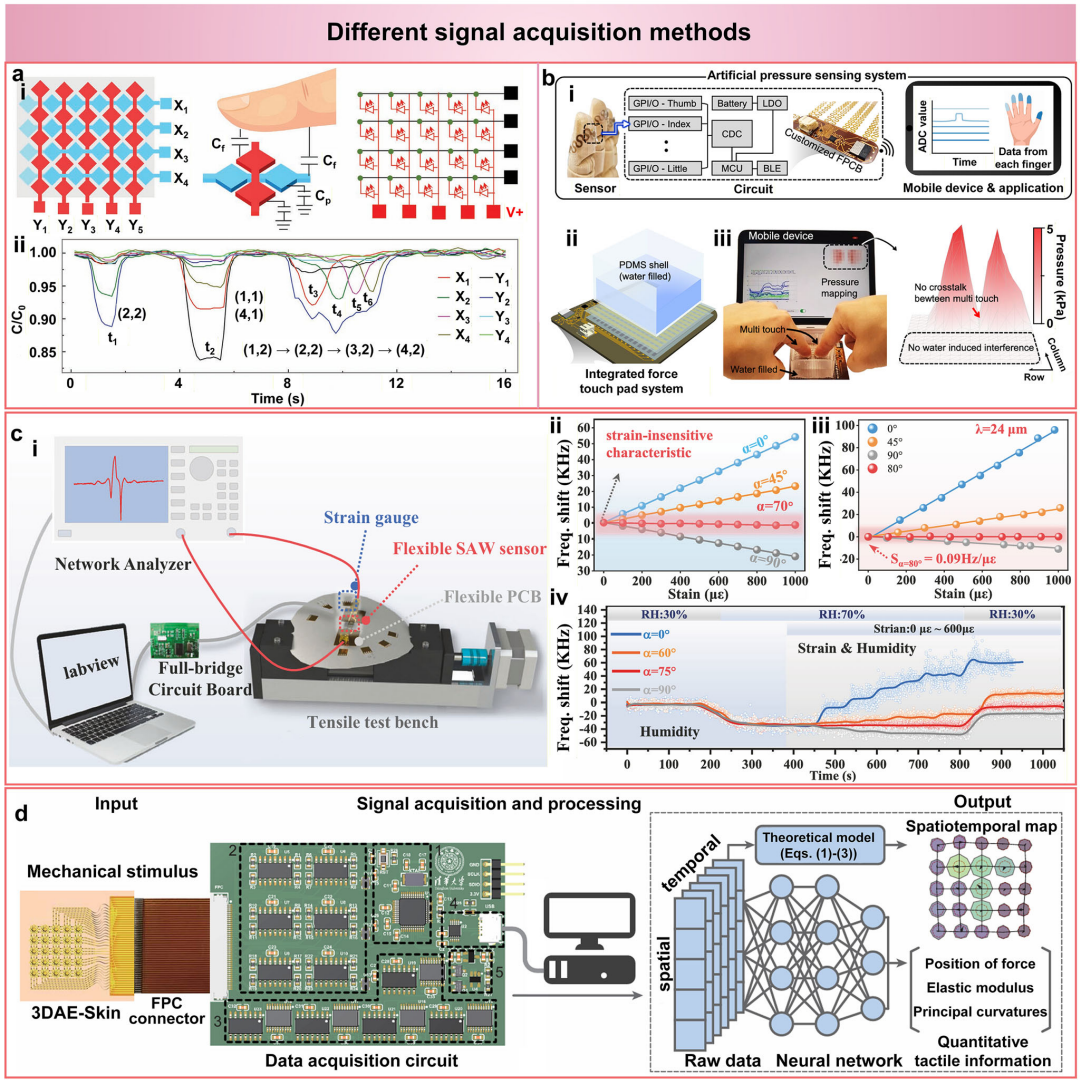
Different signal acquisition methods for soft sensors. (a) Direct electrical readout: Raw electrical signals are directly acquired from a capacitive sensing matrix. Reproduced with permission [23]. Copyright 2023, Wiley‐VCH. (i) Schematic of the sensing matrix and measurement circuit. (ii) Real‐time capacitance response (C/C_0_) from multiple sensing nodes under sequential touch. (b) Multichannel acquisition: Integrated circuit system enabling simultaneous signal collection and wireless transmission. Reproduced with permission [43]. Copyright 2025, Springer Nature. (i) Circuit configuration combining a nanogap capacitive sensor array with a customized flexible printed circuit board (FPCB), capacitance‐to‐digital converter (CDC), microcontroller unit (MCU), and Bluetooth low energy (BLE) module. (ii) Schematic of the polydimethylsiloxane (PDMS)‐based touch pad system. (iii) Real‐time pressure mapping on a mobile device showing multi‐touch operation without crosstalk or water interference. (c) Frequency‐domain readout: Flexible surface acoustic wave (SAW)‐based sensing system. Reproduced with permission [50]. Copyright 2022, Springer Nature. (i) Experimental setup integrating LabVIEW‐controlled readout. (ii, iii) Numerical and experimental verification of strain decoupling by off‐axis angle tuning. (iv) Frequency shift under combined strain and humidity conditions, confirming dual decoupling capability. (d) Artificial intelligence (AI)‐assisted signal processing: Intelligent signal acquisition framework integrating the 3D architected electronic skin (3DAE‐Skin) sensor array, high‐speed multichannel circuit, and neural network‐based processing pipeline. Mechanical stimuli are converted to digital spatiotemporal data, which are then processed through theoretical modeling and deep learning to reconstruct quantitative tactile maps, including force position, elastic modulus, and curvature. Reproduced with permission [113]. Copyright 2024, American Association for the Advancement of Science.

Direct electrical readout represents the most fundamental signal acquisition approach in soft sensors, where physical stimuli are directly converted into measurable electrical parameters such as resistance, capacitance, or voltage. The sensing matrix detects touch by monitoring capacitance variations (C/C_0_) at the intersection of row (X) and column (Y) electrodes (Figure 4a(i)) [23]. When external pressure is applied, the local capacitance changes are immediately reflected in the output signals (Figure 4a(ii)). This straightforward readout configuration provides fast response but is often limited by cross‐talk and noise interference in large‐scale arrays.

Multichannel acquisition strategies enable simultaneous signal collection from sensing units through integrated circuit modules. For example, the system combines a nanogap capacitive pressure sensor array with a customized flexible printed circuit board, including a capacitance‐to‐digital converter, a microcontroller unit, and a Bluetooth module (Figure 4b(i)) [43]. The acquired analog signals from each sensing channel are converted into digital form and wirelessly transmitted to a mobile device for real‐time pressure mapping (Figure 4b(ii)). This design allows interference‐free, high‐resolution signal acquisition across multiple channels while maintaining compact circuit integration and minimal crosstalk between sensing units (Figure 4b(iii)).

When different stimuli overlap in the time domain but remain separable in the frequency domain, extracting frequency‐domain responses becomes an efficient data acquisition strategy. The flexible surface acoustic wave (SAW) sensing system integrates a full‐bridge circuit and a network analyzer for frequency‐domain readout (Figure 4c(i)) [50]. Numerical calculations and experimental results both confirm that proper angular adjustment effectively suppresses strain coupling (Figure 4c(ii,iii)). When the flexible SAW is under bending with an off‐axis angle of 75°, the resonant frequency is only affected by humidity and remains nearly unchanged even when the flexible SAW device is bent with a strain up to 600 µε (Figure 4c(iv)), confirming its excellent dual decoupling capability for mechanical and environmental interferences.

When signals exhibit overlap in both temporal and frequency domains or when the data dimensionality becomes high, conventional time‐ or frequency‐domain analyses are no longer sufficient. AI‐assisted data acquisition and processing have thus emerged as an advanced approach. The AI‐assisted framework integrates the 3D architected electronic skin (3DAE‐Skin) sensor array with a high‐speed data acquisition circuit and deep learning‐based signal processing pipeline (Figure 4d) [113]. Mechanical stimuli applied to the sensor are collected through a flexible printed circuit interface and digitized by a multichannel acquisition module. Spatiotemporal data are then processed through a neural network trained with theoretical models to generate quantitative tactile outputs, including the position of applied force, elastic modulus, and principal curvature.

In summary, the continuous advancement of data acquisition approaches has greatly improved signal clarity, stability, and noise immunity, thereby laying a solid foundation for efficient and accurate signal decoupling discussed in this review.

## 3 Decoupling Strategies for Single Physical Signals

### 3.1 Stretching‐Insensitive Strategies

In wearable sensing applications involving substantial mechanical deformations, maintaining signal stability under tensile strain is essential to ensure accurate detection. Recent progress has yielded remarkable strain‐insensitive sensing behaviors through the use of tailored material systems and structural designs. Representative materials include LM [23], graphene [24, 25], CNTs [26], AgNWs [27, 28], PEDOT:PSS [29], Mxene [30, 31], and hydrogels [32, 33, 34]. When combined with appropriate structural engineering approaches, these materials exhibit varying levels of strain insensitivity, offering flexibility and robustness in strain‐decoupled sensing systems.

**FIGURE 5:**
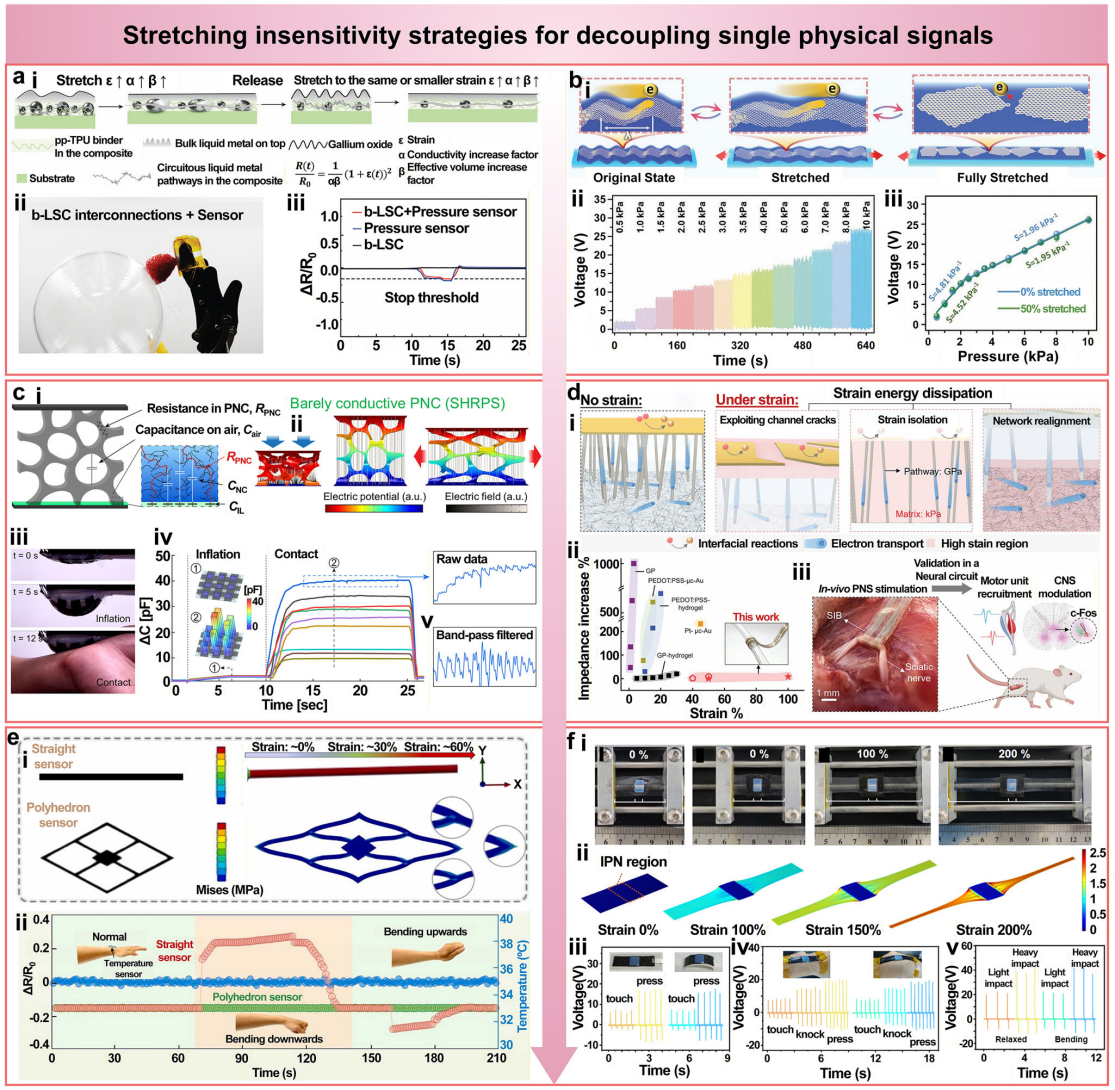
Stretching‐insensitive strategies for decoupling single physical signals. (a) Bilayer liquid‐solid conductor (b‐LSC) consisting of a wavy liquid metal top layer and a polyester polyol‐rich thermoplastic polyurethane (pp‐TPU) supported composite bottom layer, enabling dynamic reconstruction of conductive pathways under tensile strain. Reproduced with permission [23]. Copyright 2023, Wiley‐VCH. (i) Schematic illustration of the b‐LSC architecture and strain‐adaptive conduction mechanism. (ii) Application in robotic tactile sensors for fragile object handling. (iii) Real‐time resistance response to applied pressure. (b) Wrinkled graphene‐PDMS structure for strain‐insensitive pressure sensing. Reproduced with permission [24]. Copyright 2022, Wiley‐VCH. (i) Schematic of conductive channel unfolding under strain. (ii) Linear and repeatable pressure response across the range from 0.5 to 10 kPa. (iii) Comparable sensitivity retained at 0% and 50% strain. (c) PNC sensor based on carbon nanotube (CNT)‐doped Ecoflex for dual‐mode sensing. Reproduced with permission [26]. Copyright 2024, Elsevier. (i, ii) Strain‐induced capacitive dominance and pressure‐induced resistive dominance. (iii) Integration into a soft inflatable probe for real‐time pulse monitoring. (iv) Raw radial artery waveform without signal amplification. (v) Filtered waveform showing characteristic pulse peaks. (d) Strain‐isolated soft implantable bioelectronics using a tri‐layer dissipation strategy. Reproduced with permission [27]. Copyright 2022, American Association for the Advancement of Science. (i) Structural schematic showing crack‐guided interfacial layer, soft matrix, and silver nanowires (AgNW) realignment. (ii) Low impedance fluctuation under strain of up to 100%. (iii) In vivo demonstration of muscle activation and neural protein expression following sciatic nerve stimulation. (e) Polygonal PEDOT:PSS‐graphene interconnects for strain‐tolerant temperature sensing. Reproduced with permission [29]. Copyright 2024, Elsevier. (i) Simulation to compare the stress distribution in polygonal versus straight‐line routing. (ii) Stable resistance output during dynamic hand gestures. (f) Dual‐mode stretchable triboelectric nanogenerator using MXene/CNT@PDMS and UV‐induced interpenetrating polymer network. Reproduced with permission [30]. Copyright 2023, Elsevier. (i) Photographic and schematic views of the deformation. (ii) Finite element simulation showing localized strain isolation under 200% stretching. (iii, iv) Consistent pressure signals under different global strains. (v) Reliable impact sensing during joint motion from the knee and shoulder.

Owing to high electrical conductivity and exceptional mechanical compliance, LM has emerged as a promising candidate for constructing strain‐insensitive flexible conductors. A representative approach involves a bilayer liquid‐solid conductor (b‐LSC) architecture, which enhances strain tolerance by maintaining stable conductivity under large mechanical deformations [23]. In this structure, the top liquid metal layer transitions from an initially wrinkled structure to an in‐plane conducting network, effectively increasing the conductive volume. Simultaneously, the bottom elastic layer, which is composed of liquid metal particles embedded within a polyester polyol‐rich thermoplastic polyurethane (pp‐TPU) matrix, offers mechanical support and forms a secondary conductive network. As the applied strain ε increases, both the conductivity factor (α) and the effective volume factor (β) increase concurrently, collectively reducing the rate of resistance variation. The resulting resistance behavior follows $R\left(t\right)/R_{0}=\frac{1}{\alpha \beta {\left(1+\varepsilon (t)\right)}^{2}}$, which deviates significantly from theoretical predictions for conventional conductors (Figure 5a(i)), indicating that α and β jointly govern the strain‐resistance response.

This architecture has been successfully employed in flexible interconnects for robotic tactile sensing, enabling accurate pressure detection on robotic fingers (Figure 5a(ii)). A predefined resistance threshold is used to stop finger bending upon pressure detection, thus preventing damage to delicate objects such as raspberries. In the b‐LSC‐based robotic system, the strain‐insensitive characteristics ensure that finger motion does not interfere with pressure signal transmission, allowing reliable threshold recognition and effectively avoiding mechanical damage (Figure 5a(iii)).

Another representative strategy involves the use of wrinkled structures formed by embedding graphene into pre‐stretched polydimethylsiloxane (PDMS) substrates [24]. In this approach, the elastomer is first mechanically stretched to induce surface wrinkles, followed by subsequently transferring graphene sheets into the wrinkle valleys to form expandable conductive pathways. During tensile deformation, the graphene unfolds along the wrinkle direction rather than fracturing, thereby maintaining continuous electrical conduction (Figure 5b(i)). The sensor exhibits a linear and repeatable pressure response in the range from 0.5 to 10 kPa (Figure 5b(ii)). Remarkably, the device maintains comparable sensitivity at a tensile strain of 0% and 50% (Figure 5b(iii)), demonstrating robust strain‐insensitive pressure sensing capability.

In another example, a porous nanocomposite (PNC) composed of Ecoflex doped with a small amount of CNT has been developed to achieve strain‐insensitive sensing by leveraging both resistive and capacitive responses within a low‐conductivity elastomer matrix (Figure 5c(i)) [26]. Due to its inherently high resistance, the stretchable hybrid response pressure sensor (SHRPS) exhibits a primarily capacitive response under tensile strain, which is minimally influenced by stretching. In contrast, compressive deformation leads to pore collapse, resulting in a sharp decrease in resistance and a transition to a resistive‐dominated response (Figure 5c(ii)). This dual‐mode sensing behavior has been applied in a soft inflatable probe for real‐time pulse monitoring (Figure 5c(iii)), where distinct arterial waveforms from the radial artery can be captured directly without signal amplification or complex post‐processing (Figure 5c(iv)). A simple bandpass filter is sufficient to extract clear characteristic peaks from the raw signal (Figure 5c(v)).

Another effective approach involves strain isolation through layered composite structures designed to dissipate mechanical deformation in soft strain‐insensitive bioelectrodes [27]. This strategy incorporates three synergistic dissipation mechanisms: 1) an interfacial layer that relieves strain via crack propagation channels, 2) a vertical conduction path embedded in a soft matrix to isolate mechanical deformation, and 3) a bottom AgNWs network layer that dynamically reconfigures to maintain in‐plane conductivity (Figure 5d(i)). The spatial decoupling between the conductive pathway and the stress field significantly minimizes impedance fluctuation under strain, with only a 7% increase in impedance observed at a tensile strain of 100%, which is markedly superior to that of conventional stretchable materials (Figure 5d(ii)). In vivo peripheral nerve stimulation (PNS) evaluations further confirm the device's reliability, demonstrating successful muscle activation and enhanced neural protein expression in the central nervous system (CNS), as indicated by elevated c‐Fos expression following sciatic nerve stimulation (Figure 5d(iii)).

Geometric optimization also provides a practical strategy for improving strain tolerance. For instance, a temperature sensor based on a PEDOT:PSS/graphene composite adopts a polygonal interconnect design instead of conventional linear wiring to evenly distribute mechanical stress and reduce local strain concentration (Figure 5e(i)) [29]. Different from the significant signal fluctuations observed with straight‐line layouts during real‐time wearable testing, the polygonal configuration exhibits stable signals with negligibly small resistance changes across a range of hand gestures (Figure 5e(ii)), thereby confirming enhanced anti‐interference performance.

Last, a dual‐mode stretchable triboelectric nanogenerator based on the MXene/CNT@PDMS composite demonstrates effective strain isolation through localized modulus reinforcement [30]. The device incorporates an interpenetrating network (IPN) strategy, where UV‐induced crosslinking forms spatially confined high‐modulus regions within the intermediate layer. Even under the global tensile strain of 200%, deformation within the reinforced zones remains below 15% (Figure 5f(i,ii)). This structural design allows the device to maintain stable electrical output across diverse surface conditions and also to respond accurately to pressure stimuli during stretching (Figure 5f(iii,iv)). Furthermore, when mounted on highly mobile body regions such as the knee or shoulder, the device reliably captures transient impact signals associated with dynamic motion (Figure 5f(v)).

To provide a clear comparison among representative materials commonly used in stretching‐insensitive strategies, Table 1 summarizes their key performance parameters, including strain tolerance, stability under strain, and long‐term durability. Overall, these examples highlight that achieving strain insensitivity relies on balancing electrical continuity and mechanical compliance through both material and structural designs. Liquid metals and conductive composites provide excellent conductivity and elasticity but require careful interface engineering to ensure mechanical robustness, while carbon‐based and polymeric systems offer superior elasticity yet often face trade‐offs in long‐term stability. Future developments are expected to focus on hybrid structures that integrate multiple conductive phases and architectures, enabling scalable and reliable strain‐decoupled sensing for wearable applications.

**TABLE 1:** Quantitative comparison of representative stretching‐insensitive materials and their key performance parameters.

Material system	Representative structure or matrix	Strain tolerance (%)	Stability under strain	Long‐term stability (cycles)	Refs.
Liquid Metal	Bilayer liquid–solid conductor	2266	ΔR/R_0_ = 30 at 2266% strain	10 000 at 100% strain	[23]
Graphene	Wrinkled graphene composite	100	ΔR/R_0_ ≈ 2 at 100% strain	20 000 at 100% strain	[24]
CNT–Ecoflex	Barely conductive porous nanocomposite	40	ΔC_s_/ΔC_p_ = 1.8% at 40% strain	5000 at 40% strain	[26]
AgNW & ACF	Tri‐layer soft bioelectrode	100	ΔI/I_0_ ≈ 0 at 40% strain	10 000 at 20% strain	[27]
PEDOT:PSS & PVA & Graphene	Polygonal composite interconnect	40	ΔR/R_0_ ≈ 0 at 40% strain	500 at 10% strain	[29]
MXene & CNT	Dual‐mode MXene/CNT interpenetrated network	120	ΔV ≈ 0 at 120% strain	400 at 10% strain	[30]

### 3.2 Bending‐Insensitive Strategies

**FIGURE 6:**
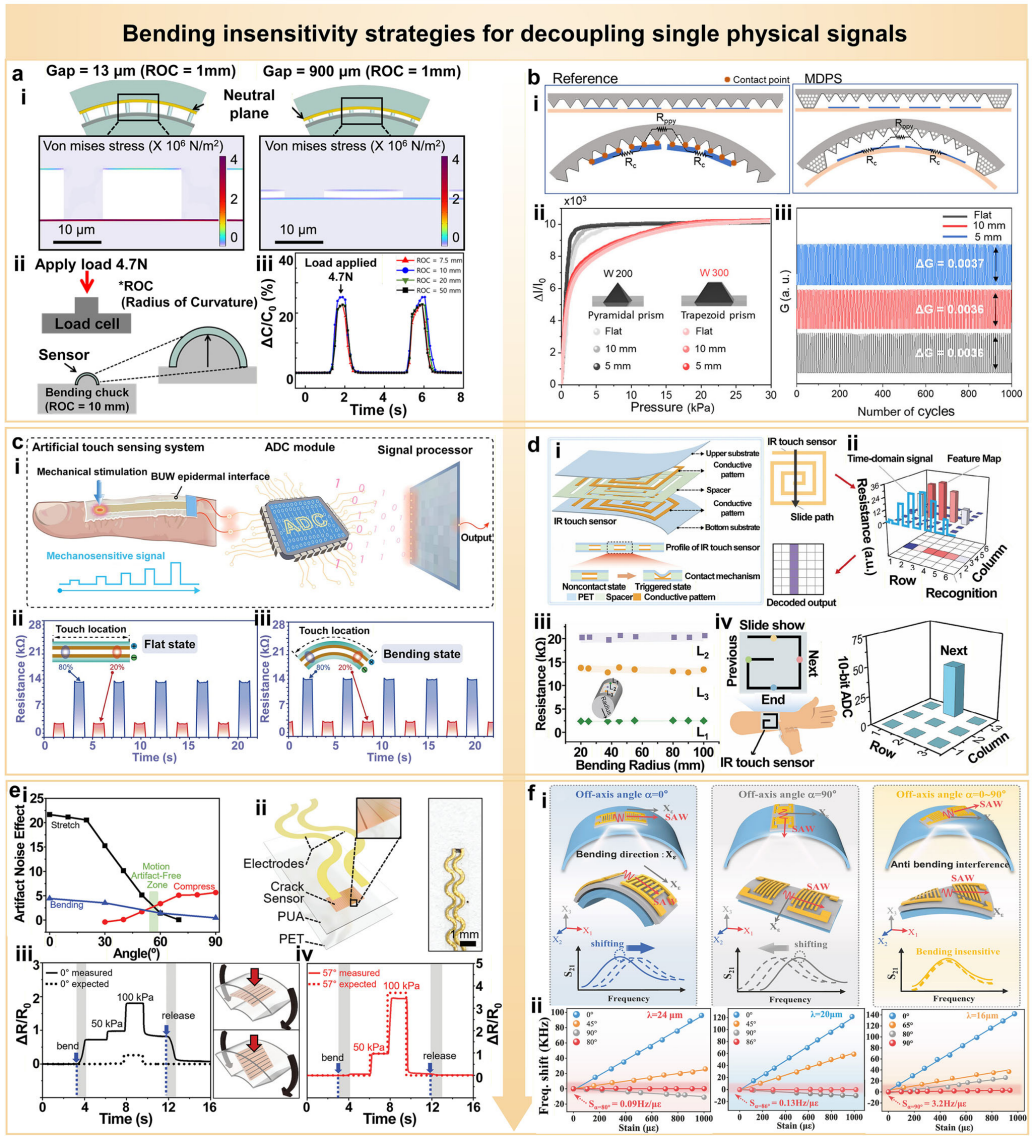
Bending insensitive strategies for decoupling single physical signals. (a) Neutral‐mechanical‐plane‐based multilayer sensor structure with nanoscale interlayer gaps for minimizing stress under bending. Reproduced with permission [43]. Copyright 2025, Springer Nature. (i) Finite element simulations comparing von Mises stress between 13 µm and 900 nm interlayer gaps under a radius of curvature (ROC) of 1 mm. (ii) Schematic of vertical load testing setup (4.7 N). (iii) Nearly identical capacitive responses under varying bending radii, confirming bending insensitivity. (b) Pressure sensor array utilizing trapezoidal prism support to avoid premature electrode contact during bending. Reproduced with permission [44]. Copyright 2022, Springer Nature. (i) Structural comparison between conventional pyramidal design and mechanically decoupled pixel sensor (MDPS). (ii) Current response consistency across flat, 10 mm, and 5 mm bending radii. (iii) Cyclic loading tests demonstrating stable conductance variation within 0.0036–0.0037. (c) CNT‐based bending‐insensitive flexible sensor using bio‐inspired upper wearable interface. Reproduced with permission [47]. Copyright 2023, Springer Nature. (i) System schematic with integrated analog‐to‐digital conversion and signal processing. (ii, iii) Reproducible signal output at 20% and 80% touch positions under both flat and bent conditions. (d) Intent recognition (IR) touch sensor architecture with integrated isolation layer and biomimetic helical web for sliding gesture detection. Reproduced with permission [48]. Copyright 2024, Wiley‐VCH. (i) Layered design with time‐domain mapping of contact events. (ii) Sliding input converted into trajectory‐specific electrical signatures. (iii) Consistent resistance responses from channels L1–L3 under bending radii from 20 to 100 mm. (iv) Real‐time interface control via sliding gesture recognition. (e) Installation‐angle‐optimized crack‐based sensor for motion artifact suppression. Reproduced with permission [49]. Copyright 2022, Wiley‐VCH. (i) Identification of minimal artifact zone near 57° installation angle. (ii) Sensor structure comprising polyethylene terephthalate (PET) substrate, polyurethane acrylate (PUA) adhesion layer, and serpentine‐patterned crack‐based sensing film. (iii, iv) Pressure response under varying installation angles with minimized bending‐induced signal deviation. (f) Directionally tuned SAW sensors for mitigating bending‐induced frequency drift. Reproduced with permission [50]. Copyright 2022, Springer Nature. (i) Schematic illustrating opposite frequency shifts under offset angles of 0° and 90°, indicating the presence of a critical compensation angle. (ii) Frequency response of SAW devices with different wavelengths (λ = 24, 20, 16 µm) under varied offset angles, confirming drift suppression at optimal alignment.

In wearable electronic systems, devices need to conform to complex human body surfaces such as joints, muscles, and the spine, where repeated bending is inevitable [35, 36, 37, 38]. Compared to uniaxial stretching, bending often induces larger local strain gradients and asymmetric structural deformation, which can lead to rupture of conductive pathways, signal drift, and baseline shift [39, 40, 41, 42]. Therefore, the development of bending‐insensitive sensor architectures is critical for achieving stable and reliable data acquisition. Recent advances have predominantly focused on structural design strategies to address this challenge. One effective approach involves symmetrically aligning the electrodes near the neutral mechanical plane and incorporating nanoscale interlayer gaps to mitigate stress interference induced by bending. Finite element simulations indicate that reducing the interlayer gap to 900 nm significantly suppresses stress concentration compared to a 13 µm‐gap design under a radius of curvature (ROC) of 1 mm, while also shifting the peak stress in the conductive layer closer to the neutral mechanical plane for minimized bending strain (Figure 6a(i)) [43]. Under a constant vertical load of 4.7 N, the device demonstrates nearly identical capacitive responses across various bending radii (Figure 6a(ii,iii)), confirming its excellent bending insensitivity.

Conventional pyramidal microstructures tend to induce unintended contact between micropillars and electrodes upon bending, resulting in abnormal variations in contact resistance (R_c_) and signal distortion [44]. To address this, a bending‐insensitive pressure sensor array adopts a design featuring trapezoidal prism structures with increased height, serving as boundary support elements to prevent premature contact (Figure 6b(i)). This architecture yields consistent and overlapping current responses under varying bending radii (i.e., flat, 10 mm, and 5 mm) across a broad pressure range (Figure 6b(ii)). Furthermore, cyclic loading tests show that the conductance variation (ΔG) remains confined within a narrow range of 0.0036–0.0037, indicating excellent structural robustness (Figure 6b(iii)).

Ultrathin CNT conductive layers also demonstrate exceptional bending insensitivity, making them well‐suited for stable signal transmission on highly curved surfaces [45, 46, 47]. In a representative implementation, a CNT‐based sensor with bending‐insensitive, unpixelated, and waterproof (BUW) characteristics converts mechanical stimuli into electrical signals through a bio‐inspired upper wearable interface, followed by analog‐to‐digital conversion (ADC) and signal processing (Figure 6c(i)) [47]. Touch inputs at 20% and 80% positions along the sensor length direction are clearly distinguishable in the unbent state (Figure 6c(ii)). More notably, identical contact positions generate nearly indistinguishable responses from the device before and after bending, confirming reliable bending‐insensitive performance (Figure 6c(iii)).

Introducing an isolation layer is another effective strategy to eliminate bending‐induced signal artifacts [48]. An intent recognition (IR) sensing architecture is realized by incorporating a gap layer between upper and lower conductive patterns, ensuring that the circuit is only activated by deliberate pressing or sliding actions (Figure 6d(i)). This configuration stabilizes the electrode contact interface under bending deformation and prevents unintended triggering. Coupled with a biomimetic helical web design, the system translates sliding motion into 2D electrical signals for trajectory recognition (Figure 6d(ii)). Across a wide bending radius range (20–100 mm), resistance variations in the three sensing channels (L1, L2, L3) remain highly consistent with minimal drift, demonstrating robust dynamic reliability (Figure 6d(iii)). This approach further enables practical interactive control tasks such as page turning and interface switching (Figure 6d(iv)).

Although crack‐based strain sensors offer high sensitivity, they are often susceptible to motion artifacts induced by dynamic bending or stretching [49]. By optimizing the mounting angle of the sensor, a minimal artifact zone can be achieved near 57°, effectively suppressing deformation‐induced noise while preserving pressure sensitivity (Figure 6e(i)). The sensor structure consists of a polyethylene terephthalate (PET) substrate, a polyurethane acrylate (PUA) adhesive layer, and a crack‐based sensing film, with serpentine electrodes incorporated to enhance mechanical compliance (Figure 6e(ii)). Among various tested orientations, the 57° configuration yields pressure signals that most accurately reflect true input values with minimal interference from bending (Figure 6e(iii,iv)).

For flexible SAW sensors, minimizing frequency drift under bending can be realized by adjusting the offset angle between the acoustic wave propagation direction and the axis of mechanical deformation (Figure 6f(i)) [50]. When the offset angle α is 0° or 90°, bending induces opposite frequency shifts, indicating the existence of a compensatory angle. Frequency response measurements of SAW devices with different wavelengths (λ = 24, 20, and 16 µm) under various offset angles confirm this trend (Figure 6f(ii)). Notably, the device with λ of 24 µm exhibits the lowest strain sensitivity (0.09 Hz/µε) at α = 80°, while the device with λ of 16 µm continues to exhibit residual drift even at α = 90°, suggesting that offset‐angle optimization must be co‐designed with the acoustic wavelength.

In summary, these strategies, ranging from electrode alignment and microstructure isolation to mechanical compliance engineering and directional optimization, collectively enable the development of bending‐insensitive sensor architectures. From a practical standpoint, future progress should focus on simplifying structural designs and improving compatibility with large‐area fabrication processes. For instance, replacing complex multilayer assemblies with ultrathin composite films or printed electrode configurations could significantly reduce structural complexity while maintaining bending stability. Moreover, integrating these mechanical strategies with circuit‐level compensation and calibration algorithms may further ensure reliable data acquisition during continuous or multidirectional deformation, paving the way for scalable bending‐insensitive systems.

### 3.3 Other Insensitive Strategies

While tensile and bending deformations are among the most prevalent sources of signal interference in flexible sensors, real‐world applications also encounter disturbances induced by non‐target environmental factors such as temperature [51, 52, 58, 89], humidity [53, 54, 59], gas [55, 56], pressure [60], and ambient light [57, 61]. To ensure sensing accuracy under complex conditions, various targeted suppression strategies have been exploited.

**FIGURE 7:**
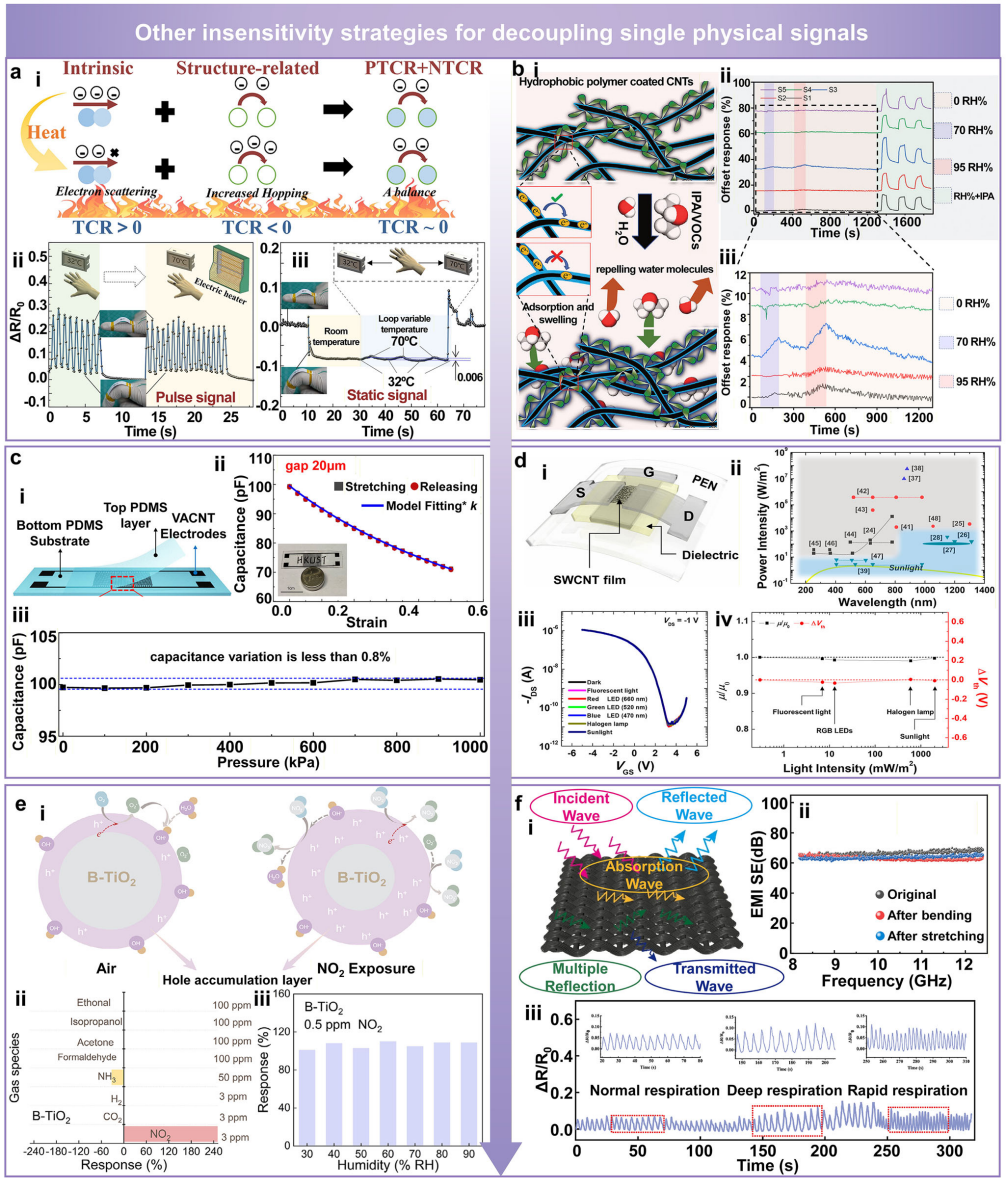
Other insensitive strategies for decoupling single physical signals. (a) Temperature‐insensitive strain sensing based on the balance between positive and negative temperature coefficient resistance (PTCR/NTCR) effects. Reproduced with permission [58]. Copyright 2023, Elsevier. (i) Schematic of electron scattering and inter‐particle carrier hopping mechanisms. (ii) Stable resistance output under cyclic strain at 32°C and 70°C. (iii) Negligible resistance drifts under static loading with temperature variations. b) Humidity‐insensitive sensing achieved via hydrophobic polymer coating on CNT. Reproduced with permission [59]. Copyright 2025, Wiley‐VCH. (i) Illustration of moisture exclusion and VOC selectivity. (ii) Consistent signal response under 0%, 70%, and 95% relative humidity (RH) and isopropanol exposure. (iii) Enlarged view highlighting minimal channel‐to‐channel signal deviation. (c) Pressure‐insensitive capacitive sensor using a vertically aligned CNT (VACNT)‐based 3D interdigitated electrode structure. Reproduced with permission [60]. Copyright 2022, Springer Nature. (i) Device architecture embedded in the PDMS matrix. (ii) Highly reversible capacitive strain response. (iii) Strong pressure insensitivity with less than 0.8% capacitance change under 0–1 MPa pressure. (d) Illumination‐insensitive thin‐film transistor based on high‐purity single‐walled carbon nanotubes (SWCNT). Reproduced with permission [61]. Copyright 2023, American Chemical Society. (i) Device configuration using polyethylene‐naphthalate (PEN) substrate and thermally evaporated metal electrodes. (ii) Absence of significant photoresponse under typical ambient light. (iii) Overlapping transfer characteristics across various lighting conditions. (iv) Stable field‐effect mobility (µ/µ_0_) and threshold voltage (ΔVth) to confirm light insensitivity. (e) Gas‐insensitive sensing based on black TiO_2_ (B‐TiO_2_) with selective NO_2_ detection and strong humidity tolerance. Reproduced with permission [62]. Copyright 2025, Wiley‐VCH. (i) Schematic of the sensing mechanism showing oxygen adsorption in air and enhanced hole concentration under NO_2_ exposure. (ii) Selectivity of B‐TiO_2_ toward various gases, showing a dominant response to NO_2_. (iii) Stable response to 0.5 ppm NO_2_ under 30%–90% RH. (f) Electromagnetic interference (EMI)‐insensitive sensing using a superhydrophobic MXene composite. Reproduced with permission [63]. Copyright 2023, Springer Nature. (i) Schematic of multiple reflection, absorption, and transmission of incident waves. (ii) EMI shielding effectiveness (SE) above 60 dB maintained under bending and stretching. (iii) Stable resistance response under different breathing patterns.

For mitigating temperature‐induced interference, one representative approach involves the synergistic tuning of positive temperature coefficient (PTCR) and negative temperature coefficient (NTCR) behaviors within the conductive network, enabling temperature‐insensitive performance across a broad temperature range (Figure 7a(i)) [58]. In this design, graphene nanoplatelets exhibit PTCR characteristics due to increased electron scattering at elevated temperatures, whereas inter‐particle tunneling pathways show NTCR behavior as a result of enhanced carrier hopping. By optimizing the ratio and spatial distribution of these effects, the overall resistance response can be effectively stabilized against temperature fluctuations. Experimental results validate that the sensor maintains a stable resistance output with negligible variation under cyclic strain loading at 32°C and 70°C (Figure 7a(ii)). Moreover, the device demonstrates excellent thermal stability during static loading, with a temperature‐induced resistance change rate as low as 0.006 (Figure 7a(iii)).

To address humidity‐induced interference, a hydrophobic polymer coating is applied to the surface of single‐walled carbon nanotubes (SWCNT) to suppress water molecule adsorption and associated swelling effects (Figure 7b(i)) [59]. This coating layer functions as a moisture barrier, preventing the ingress of water into the conductive CNT network and thereby maintaining pathway stability. Notably, the structure selectively responds to volatile organic compounds, while remaining inert to humidity variations. Performance evaluations under 0%, 70%, and 95% relative humidity (RH), as well as in the presence of isopropanol, confirm the high stability of the functionalized SWCNT array (Figure 7b(ii)), with negligible signal deviation across channels (Figure 7b(iii)) to validate its humidity‐insensitive characteristics.

To mitigate pressure‐related interference, a 3D interdigitated electrode architecture based on vertically aligned carbon nanotubes (VACNT) has been developed (Figure 7c(i)) [60]. In this design, VACNT arrays are sandwiched between upper and lower PDMS layers, forming a rigid vertical structure. During tensile deformation, electrode spacing changes in a controlled manner, yielding highly predictable and reversible capacitive responses (Figure 7c(ii)). Since the electrode alignment is perpendicular to the direction of applied pressure and the structure maintains high vertical rigidity, the inter‐electrode distance remains nearly constant under compression. Consequently, capacitive variation is less than 0.8% within a pressure range of 0–1 MPa, demonstrating excellent pressure insensitivity (Figure 7c(iii)).

To suppress optical interference, flexible thin‐film transistors have been constructed using high‐purity SWCNT films as the active channels, along with polyethylene‐naphthalate (PEN) substrates and thermally evaporated metal electrodes (Figure 7d(i)) [61]. Due to the 1D electronic structure of SWCNT and their large exciton binding energy, low‐energy photons from typical ambient light sources are insufficient to generate free carriers, rendering the device intrinsically robust against visible light exposure. Optical characterization reveals that photoresponse in SWCNT occurs only under specific wavelengths and at high power densities, far exceeding those encountered under ambient conditions (Figure 7d(ii)). Experimental measurements under fluorescent lighting, RGB LEDs, halogen lamps, and natural sunlight show nearly overlapping transfer curves with no observable drift in the output current (Figure 7d(iii)). In addition, the field‐effect mobility (µ/µ_0_) and threshold voltage shift (ΔV_th_) remain stable across varying lighting conditions, confirming the device's illumination‐insensitive characteristics in practical environments (Figure 7d(iv)).

To achieve high gas selectivity, a black TiO_2_ (B‐TiO_2_)‐based sensor was developed for the selective detection of oxidizing gases such as NO_2_ under ambient conditions (Figure 7e(i)). [62] Upon exposure to air, oxygen adsorption on the p‐type B‐TiO_2_ surface extracts electrons and forms a hole accumulation layer. When exposed to NO_2_, the strong electron‐withdrawing ability of the gas further increases the hole concentration, resulting in a pronounced resistance change. In contrast, reducing gases such as NH_3_ and ethanol induce negligible charge transfer, leading to minimal response. The bar chart (Figure 7e(ii)) demonstrates the outstanding selectivity of B‐TiO_2_ toward NO_2_ compared with other interfering gases including H_2_, CO_2_, NH_3_, and volatile organics. Furthermore, the response to 0.5 ppm NO_2_ remains nearly unchanged across 30%–90% relative humidity (Figure 7e(iii)), confirming the humidity‐insensitive nature of B‐TiO_2_ arising from its suppressed water adsorption and electronic‐dominated conduction.

To address electromagnetic interference (EMI) and ensure reliable operation in dynamic environments, a superhydrophobic MXene‐based fabric was constructed to achieve reflection‐dominated EMI shielding and stable signal transmission (Figure 7f(i)) [63]. In this configuration, incident electromagnetic waves are largely reflected by the highly conductive MXene network, while a small portion is absorbed through interfacial polarization and multiple internal reflections, yielding an overall shielding effectiveness (SE) exceeding 66 dB. Experimental characterization confirms that the EMI SE of this fabric remains nearly unchanged after 500 bending and stretching cycles (Figure 7f(ii)), demonstrating excellent mechanical robustness and electrical stability under deformation. Beyond electromagnetic protection, the conductive textile functions as a wearable strain sensor capable of capturing subtle respiratory signals (Figure 7f(iii)). Distinct ΔR/R_0_ waveforms corresponding to normal, deep, and rapid breathing states are clearly identified, validating its capability for continuous respiratory monitoring with high sensitivity and real‐time reliability.

In summary, by tailoring electrical properties, introducing physical barrier layers, incorporating directionally selective structures, and engineering surface chemistry or conductive networks, sensors can achieve robust, accurate, and stable performance even in the presence of diverse non‐target environmental stimuli. Although considerable progress has been made in decoupling single environmental interferences, real‐world environments often involve concurrent variations that lead to coupled effects on sensing performance. To address such multiplex interference, developing hybrid coatings that integrate materials with distinct thermal and hygroscopic properties, such as hydrophobic polymers, inorganic oxides, and temperature‐insensitive fillers, could be a promising strategy. For example, an optical fiber‐based gas sensor employing a chitosan/PDMS hybrid coating effectively exemplifies this concept [64]. The hydrophilic chitosan layer provides strong chemical affinity toward formic acid molecules through hydrogen bonding, while the outer hydrophobic PDMS layer suppresses moisture adsorption and minimizes humidity‐induced refractive index variations. This bilayer configuration enables the simultaneous detection of formic acid, temperature, and humidity, with a data‐driven correction model to decouple thermal and hygroscopic interferences. The resulting system achieves high sensitivity, selectivity, and environmental robustness. Such hybrid architectures are expected to provide an exciting pathway for decoupling multiple environmental stimuli, enabling reliable and interference‐resistant sensing performance under complex real‐world conditions.

### 3.4 Crosstalk‐Free Strategies for Sensor Arrays

As flexible sensors evolve from single‐unit devices into array‐based systems, additional challenges emerge beyond conventional mechanical and environmental interferences. One of the most critical issues at the array level is inter‐channel crosstalk [65, 66, 67, 68, 69, 70, 71]. When multiple sensing pixels are simultaneously stimulated, the absence of effective structural isolation or signal decoupling mechanisms can result in mutual interference among adjacent units, ultimately compromising spatial resolution and recognition accuracy. To ensure high‐fidelity, pixel‐level signal acquisition, both device architecture and circuit design must be carefully engineered.

**FIGURE 8:**
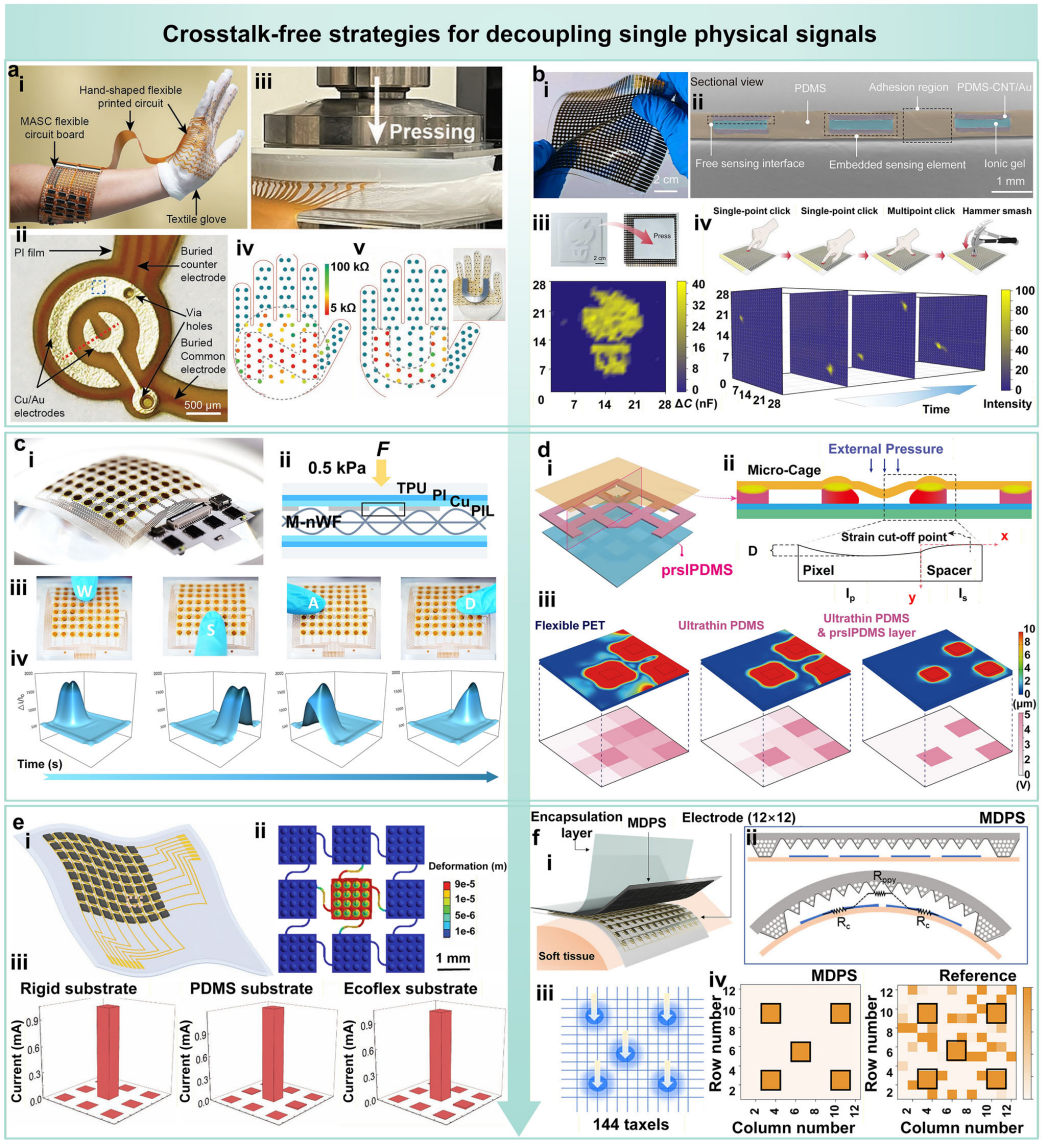
Crosstalk‐free strategies for decoupling single physical signals in flexible sensor arrays. (a) Flexible hand‐shaped circuit board integrated with 112 bimodal sensing units for pressure and flexion detection. Reproduced with permission [72]. Copyright 2022, Wiley‐VCH. (i, ii) Embedded electrode structure beneath a polyimide (PI) layer. (iii, iv) Softness mapping of composite‐layered materials with distinct moduli (soft: 89 kPa; hard: 1.0 MPa). (v) Accurate multipoint recognition using a U‐shaped object without inter‐channel interference. (b) Ionic skin array with isolated microstructured ionic gels (IMIGs) embedded within a perforated PDMS substrate. Reproduced with permission [73]. Copyright 2023, American Association for the Advancement of Science. (i) Device layout showing individually compartmentalized ionic channels. (ii) Sectional view demonstrating pixel‐level ionic isolation. (iii) High‐resolution pressure mapping under localized loading. (iv) Distinct signal responses under various contact scenarios. (c) Pressure sensor array employing micropore PI isolation layer (PIL), MXene‐coated nonwoven fabric (M‐nWF), and serpentine PI/Cu interconnects. Reproduced with permission [74]. Copyright 2024, Wiley‐VCH. (i) Device configuration illustrating structural isolation. (ii) Robust signal stability under stretching and bending due to mechanical isolation. (iii) Accurate multipoint recognition performance. (iv) No false triggering observed in unloaded regions. (d) Spatially localized pixel design using photo‐reticulated strain localization PDMS (prslPDMS). Reproduced with permission [75]. Copyright 2023, Springer Nature. (i) Schematic of the microcage isolation structure. (ii) Effective strain cut‐off at pixel boundaries prevents stress propagation. (iii) Comparative analysis showing lower crosstalk than conventional PET or PDMS isolation layers. (e) High‐resolution laser‐induced graphene (LIG)‐based sensor array with serpentine interconnects for mechanical strain isolation. Reproduced with permission [76]. Copyright 2022, Wiley‐VCH. (i) Structural overview of multilayer design. (ii) Reduced mechanical coupling between pixels using serpentine layout. (iii) Consistent single‐point signal response across rigid, PDMS, and Ecoflex substrates. (f) MDPS array integrated with high‐aspect‐ratio trapezoidal prism supports. Reproduced with permission [44]. Copyright 2022, Springer Nature. (i) Flexible system‐level integration scheme. (ii) Cross‐sectional view of MDPS under bending. (iii) Localized activation in a 12 × 12 array (144 sensing units) under multipoint input. (iv) Clear five‐point contact recognition with significantly reduced crosstalk compared to conventional array design.

A representative example involves a flexible hand‐shaped circuit board integrating 112 bimodal sensing units for high‐density detection of pressure and joint flexion across the human hand (Figure 8a(i)) [72]. Each sensing pixel comprises a grounded electrode embedded beneath a polyimide (PI) layer and a corresponding top electrode (Figure 8a(ii)). The embedded configuration, along with insulating dielectric layers, effectively suppresses electrical crosstalk between neighboring channels. Under asymmetric mechanical loading such as 89 kPa on the upper and 1 MPa on the lower surfaces, the array maintains excellent channel isolation, with minimal signal interference (Figure 8a(iii,iv)). During multi‐point recognition tests using a U‐shaped contact object, the system accurately reconstructs the contact geometry with a sharp boundary and negligible inter‐pixel leakage (Figure 8a(v)).

Another effective approach involves embedding isolated microstructured ionic gels within a perforated PDMS substrate to construct a flexible ionic skin array with a resolution of up to 28 × 28 pixels (Figure 8b(i)) [73]. The physical separation of ionic pathways between individual sensing units significantly reduces crosstalk compared to traditional architectures utilizing continuous ionic layers (Figure 8b(ii)). Under localized pressure, signal interference in adjacent pixels remains below 0.02%. Furthermore, a dedicated readout circuit incorporating a crosstalk compensation algorithm enhances detection accuracy, enabling high‐resolution pressure mapping (Figure 8b(iii)). The array reliably reconstructs input patterns and accurately localizes pressure points under various loading scenarios, including single‐point, multipoint, and dynamic impact, without observable signal distortion (Figure 8b(iv)).

Structural isolation continues to serve as a core strategy for crosstalk suppression. A pressure sensor array has been developed using micropore PI isolation layers (PIL) and serpentine‐shaped PI/Cu interconnects, integrated with an MXene‐coated nonwoven fabric (M‐nWF) sensitive layer (Figure 8c(i)) [74]. The porous isolation layer acts as a mechanical buffer, dispersing external forces and preventing localized stress concentration, while the serpentine electrode layout minimizes mechanical coupling across neighboring pixels. This design enables crosstalk‐free signal acquisition even under stretching and bending conditions (Figure 8c(ii)). The array accurately detects and differentiates multiple contact points during simultaneous multipoint pressing (Figure 8c(iii)), while maintaining zero response in non‐contacted regions (Figure 8c(iv)).

To further enhance spatial isolation between pixels, a photo‐reticulated strain‐localization PDMS (prslPDMS) microcage architecture has been introduced (Figure 8d(i)) [75]. In this design, each sensing unit is encapsulated within an individually confined cavity formed by the prslPDMS framework, effectively restricting mechanical deformation from propagating across neighboring pixels (Figure 8d(ii)). Compared to conventional isolation layers composed of PET or unstructured PDMS, the prslPDMS design exhibits markedly reduced inter‐pixel interference under multipoint loading conditions (Figure 8d(iii)).

Laser‐induced graphene (LIG) technology has also been utilized to fabricate flexible sensor arrays with high spatial resolution within multilayer polymer stacks (Figure 8e(i)) [76]. The incorporation of serpentine interconnects effectively mitigates mechanical strain transfer between adjacent sensing units, thereby preventing false triggering often observed in conventional straight‐line layouts under localized compression (Figure 8e(ii)). The LIG‐based array demonstrates robust single‐point discrimination and excellent crosstalk immunity across various substrate materials, including rigid films, PDMS, and Ecoflex (Figure 8e(iii)).

The previously discussed mechanical decoupling strategy based on high‐aspect‐ratio structural supports, such as trapezoidal prism arrays [44], has also been adapted for sensor array integration (Figure 8f(i,ii)). In this configuration, localized contact selectively activates the corresponding sensing pixel, enabling accurate tactile imaging using a 12 × 12 array composed of 144 sensing units (Figure 8f(iii)). In a five‐point contact test, the mechanically decoupled pixel sensor (MDPS) array responds exclusively to the intended contact locations, whereas traditional designs exhibit widespread false signals across non‐target regions, indicating significant inter‐channel crosstalk (Figure 8f(iv)).

Table 2 summarizes representative array decoupling strategies on scalability‐related metrics, including array size, pixel density, and signal latency. Based on these comparisons, current technologies have enabled the integration of large sensor arrays within relatively small areas. However, further scaling is mainly limited by the increasing complexity of interconnect networks and signal acquisition circuits. Future developments should focus on reducing wiring density and minimizing the size of readout circuits to achieve higher scalability and integration efficiency.

**TABLE 2:** Comparison of scalability‐related performance metrics in crosstalk‐free strategies.

Strategy	Typical array size	Pixel density	Response latency	Representative refs.
Common‐grounded array	10 × 20	20 × 20 units on 12 × 23.5 cm^2^	N/A	[72]
Isolated sensing units	28 × 28	28 × 28 units on 10 × 10 cm^2^	< 5 ms	[73]
Micropore isolation layer	8 × 8	8 × 8 units on 7 × 7 cm^2^	< 70 ms	[74]
Microcage architecture	6 × 6	6 × 6 units on 2 × 2 cm^2^	< 284 ms	[75]
Serpentine interconnects	8 × 8	8 × 8 units on 2 × 2 cm^2^	< 20 ms	[76]
Trapezoidal prism design	12 × 12	12 × 12 units on 6.5 × 6.5 cm^2^	< 12 ms	[44]

## 4 Decoupling Strategies for Multimodal Physical Signals

### 4.1 Spatiotemporal Decoupling Strategies

Beyond improving robustness against individual physical interferences such as stretching, bending, temperature, and humidity, flexible sensors designed for multimodal applications must also address the more complex challenge of signal coupling arising from concurrent, intertwined stimuli. Decoupling different physical inputs has thus become a critical design objective to enhance sensing accuracy, functional differentiation, and environmental adaptability. Recent efforts have explored effective strategies for multimodal physical signals decoupling based on structural and material designs [77, 78, 79, 80, 81, 94], signal types [82, 83, 84, 85, 86, 87, 88, 89, 95], operational conditions [90], response times [91], and advanced algorithms [92, 93].

Among structure and material design strategies, a notable example is inspired by the zonal microstructure of cartilage tissue. A 3D elastic network composed of interpenetrating soft polyacrylamide (PAAm) chains and rigid cellulose nanofiber (CNF) chains has been developed to differentiate between tensile and compressive strains [94].

**FIGURE 9:**
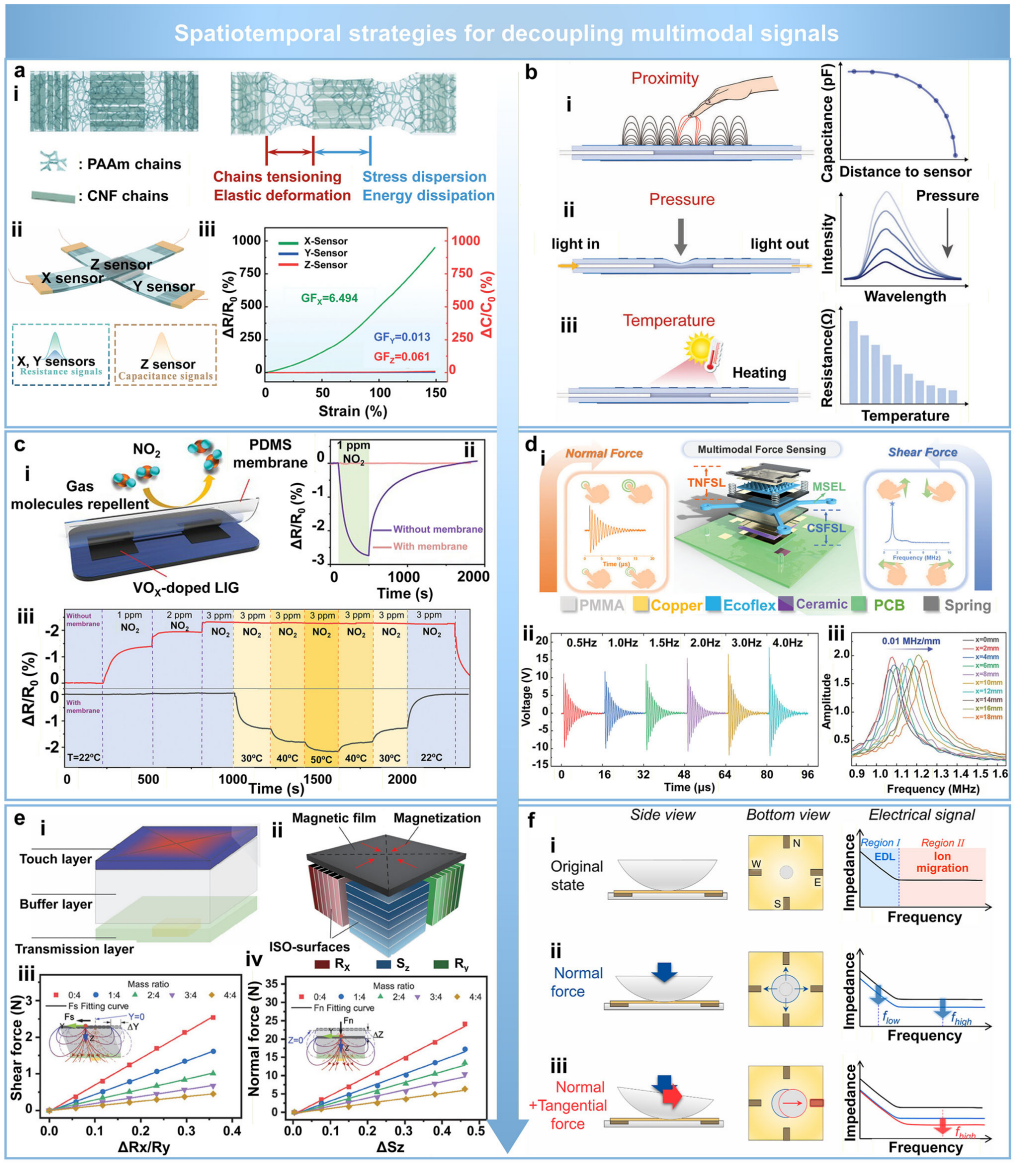
Spatiotemporal strategies for decoupling multimodal physical signals. (a) Direction‐selective 3D elastic network for discriminating tensile and compressive strains. Reproduced with permission [94]. Copyright 2024, Wiley‐VCH. (i) Structural design composed of interpenetrating soft polyacrylamide (PAAm) and rigid cellulose nanofiber (CNF) chains to achieve anisotropic stress distribution. (ii) Triaxial sensor configuration enabling simultaneous resistance and capacitance acquisition along X/Y/Z directions. (iii) Comparison of gauge factors along three directions under 150% strain, confirming directional sensitivity. (b) Physical mechanism‐based decoupling using multiple independent sensing modes. Reproduced with permission [95]. Copyright 2023, Wiley‐VCH. (i) Capacitance variation induced by proximity through fringe field disturbance. (ii) Pressure sensing via deformation of the optical waveguide and corresponding intensity attenuation. (iii) Temperature sensing based on resistance change of PEDOT:PSS due to carrier mobility modulation. (c) Functional signal decoupling through material selection and targeted encapsulation. Reproduced with permission [90]. Copyright 2023, Wiley‐VCH. (i) VO_x_‐doped LIG sensor encapsulated with PDMS to allow thermal conduction while blocking gas permeation. (ii) Encapsulation effectively suppresses NO_2_ response while preserving thermal sensitivity. (iii) Comparative response of encapsulated and unencapsulated devices to NO_2_ and temperature stimuli. (d) Signal‐domain decoupling enabled by a multilayer heterostructure with mechanical isolation. Reproduced with permission [102]. Copyright 2024, Wiley‐VCH. (i) Schematic of the device integrating triboelectric and capacitive sensing units separated by an Ecoflex buffer layer. (ii) Time‐domain voltage amplitude modulation under normal force. (iii) Wireless frequency‐domain signal under different shear displacements (0–18 mm). (e) Magnetic‐directional decoupling strategy using a split magnetic film and 3D Hall sensor. Reproduced with permission [103]. Copyright 2024, Wiley‐VCH. (i, ii) Device structure featuring centripetally magnetized micro‐magnet embedded in elastomer. (iii, iv) Independent detection of shear (Rx/Ry) and normal (Sz) forces, with tunable stiffness‐dependent response profiles. (f) Frequency‐domain decoupling using impedance spectroscopy. Reproduced with permission [104]. Copyright 2023, American Chemical Society. (i) Schematic showing dual impedance mechanisms: low‐frequency electrical double layer (EDL) and high‐frequency ionic migration. (ii) Impedance drops in both regions under pure normal force. (iii) Under combined loading, significant impedance reduction observed only in the high‐frequency region, confirming direction‐specific separation.

Laterally aligned CNF chains primarily absorb and dissipate tensile stress, while vertically oriented domains exhibit minimal deformation in the Z‐direction, thus enabling directional divergence of mechanical stress within the bulk structure (Figure 9a(i)). Leveraging this anisotropic mechanical behavior, a tri‐axial (X/Y/Z) sensing platform has been constructed to simultaneously capture resistive and capacitive signals for functionally decoupling tensile and compressive inputs (Figure 9a(ii)). Experimental results show that the X‐axis sensor exhibits the highest gauge factor (GF_x_ = 6.494) under 150% tensile strain, whereas the Y and Z axes exhibit significantly lower GF values (0.013 and 0.061, respectively), confirming the structural anisotropy and effective directional decoupling (Figure 9a(iii)). This work demonstrates that directional stress distribution can be effectively controlled through anisotropic structural design, enabling selective decoupling of tensile and compressive inputs. Such structural anisotropy can be extended beyond wearable systems to robotic tactile platforms by tuning the stiffness contrast and interfacial adhesion. Nevertheless, this stacked design increases the sensor's size in 3D space, and future studies should focus on integrated fabrication techniques to enable large‐scale and arrayed manufacturing.

For signal‐type‐based decoupling strategies, one effective approach enables functional separation of multiple inputs by employing independent and non‐interfering sensing mechanisms for each stimulus. In one representative design, proximity, pressure, and temperature are encoded via changes in capacitance, optical intensity, and resistance, respectively [95]. Specifically, an approaching finger perturbs the fringing field of interdigitated electrodes, resulting in a measurable decrease in capacitance (Figure 9b(i)). In comparison, the applied pressure compresses an optical waveguide, increasing optical loss and reducing output intensity (Figure 9b(ii)). Additionally, temperature variations modulate carrier mobility in PEDOT:PSS electrodes, leading to resistance changes (Figure 9b(iii)). The integration of orthogonal sensing domains within a single architecture enables real‐time, independent acquisition of multiple physical signals with minimal interference.

Targeted encapsulation combined with controlled operating conditions has also been proven effective for functional signal decoupling [90]. A VO_x_‐doped LIG sensor has been developed for multi‐parameter sensing, wherein a PDMS encapsulation layer selectively permits thermal conduction while effectively blocking the diffusion of gas molecules (Figure 9c(i)). Experimental results reveal that the encapsulated sensor exhibits negligible response to 1 ppm NO_2_ gas (Figure 9c(ii)), while maintaining a stable and linear response to temperature variations in the range from 30°C to 50°C (Figure 9c(iii)). In contrast, the unencapsulated sensor shows strong NO_2_ sensitivity, but exhibits limited temperature response when self‐heated to 50°C. This approach, combining physical shielding with operational control, achieves effective decoupling of gas and thermal signals.

Beyond separating mechanical, thermal, and chemical signals, another critical direction in flexible multimodal sensing involves the decoupled recognition of multidirectional mechanical inputs, such as normal and shear forces applied in orthogonal spatial orientations [96, 97, 98, 99, 100, 101, 102, 103, 104, 113]. One representative strategy achieves signal‐domain separation through the design of multilayer structures comprising heterogeneous material stacks [102]. A laminated architecture composed of polymethyl methacrylate (PMMA), copper, Ecoflex, ceramic, and printed circuit board (PCB) layers incorporates two independent sensing modules: a triboelectric nanogenerator‐based normal force sensing layer (TNFSL) at the top and a capacitive shear force sensing layer (CSFSL) at the bottom (Figure 9d(i)). The middle stretchable Ecoflex layer (MSEL) acts as a mechanical decoupling medium, preventing mode coupling between the two channels. Normal forces are captured via voltage amplitude fluctuations in the time domain, which correlate positively with the frequency of the applied load (Figure 9d(ii)), whereas shear forces are identified by analyzing frequency‐domain characteristics. Specifically, the signal exhibits different characteristic frequencies across varying contact conditions (Figure 9d(iii)), demonstrating two highly decoupled sensing modalities. While this design offers a promising route for low‐power and wireless multimodal tactile perception, its multilayer configuration and rigid ceramic components restrict conformability and large‐area scalability in both wearable and robotic applications. Future work should focus on integrating flexible circuitry and employing thin‐film dielectric materials to improve adaptability across different sensing platforms.

Magnetic‐directional decoupling represents another effective strategy for spatially resolved force detection in three dimensions [103]. A split‐type architecture integrates a magnetic film with a 3D Hall sensor, while a centripetally magnetized composite micro‐magnet embedded in an Ecoflex matrix serves as the deformable transduction medium (Figure 9e(i,ii)). Shear (Rx/Ry) and normal forces (Sz) induce directional changes in orthogonal magnetic field components, enabling accurate differentiation of force vectors. Experimental results demonstrate that tuning the stiffness of the elastomer matrix allows precise control over the force‐displacement relationship for both shear and normal inputs (Figure 9e(iii,iv)), resulting in high‐resolution, low‐error tri‐axial force measurement.

Impedance spectral analysis also offers a robust method for decoupling normal and tangential forces based on frequency‐domain response characteristics [104]. In this approach, low‐frequency signals (Region I) reflect the behavior of the electrical double layer (EDL), while high‐frequency signals (Region II) are dominated by bulk ion migration (Figure 9f(i)). Under purely normal loading, impedance reductions are observed in both regions simultaneously (Figure 9f(ii)). In contrast, combined normal and shear loads result in a pronounced impedance drop only in the high‐frequency region (Figure 9f(iii)). By comparing these distinct spectral signatures, the directionality of mechanical stimuli can be effectively resolved. Although this sensor can effectively distinguish between pressure and shear forces, its functionality could be further extended by enabling simultaneous differentiation of multiple mechanical stimuli. In particular, the quantitative detection of torque or complex stress states under severe bending or stretching conditions remains an important challenge for future research.

In addition to the above strategies, signal decoupling can also be achieved through a combination of structural design and algorithmic processing. For example, by constructing a patterned metal layer with cavity and protrusion structures on a flexible substrate, the metal layer beneath the protrusion (T‐unit) is designed to remain nearly undeformed under external pressure, making it sensitive only to temperature, while the metal layer near the cavity edges (P‐unit) responds to both pressure and temperature. Based on the dual‐channel outputs, a mathematical model is further established to separate temperature signals from pressure signals, thereby realizing pressure‐temperature decoupling [93]. Inspired by the cooperative regulation of thermoreceptors and mechanoreceptors in human skin, a bioinspired ionic‐channel modulation strategy is developed to decouple temperature and pressure signals in ionogel sensors. Through the synergistic design of densely packed and ultrathin architectures, the ionic pathways remain stable under compression while responding to temperature variations. Combined with a PEG‐400‐based pressure‐sensitive layer and mathematical modeling, this approach achieves structural‐material‐algorithmic decoupling, effectively suppressing temperature‐pressure coupling and enabling skin‐like sensing [82].

Decoupling can also be achieved by exploiting the different response times of sensors to various physical stimuli. For example, an interlocked P(VDF‐TrFE) microstructured device allows self‐decoupling of pressure and temperature signals. The sensor generates a rapid triboelectric response under pressure and a slower pyroelectric response under thermal stimulation. The distinct temporal characteristics of these two signals allow them to be clearly distinguished within a single sensing unit, enabling multimodal perception [81].

In addition, a frequency‐response‐based decoupling strategy has been proposed, in which a resistive strain unit and a capacitive pressure unit are integrated within a single flexible sensor. By employing multi‐band notch filtering, the strain and pressure signals are separated in the frequency domain. This method enables simultaneous measurement of both parameters through only two connecting wires, significantly simplifying the wiring while achieving high‐precision signal decoupling [72].

In summary, decoupling strategies based on structural and material design, signal type, operational conditions, response times, and algorithms provide a comprehensive foundation for the development of high‐throughput, multidimensional, and multimodal flexible sensing platforms. While each approach enhances sensing accuracy and functional independence from different perspectives, their applicability across wearable and robotic systems remains constrained by structural complexity and circuit integration. Future efforts should focus on unified design frameworks that couple mechanical, electrical, and computational decoupling to realize scalable, low‐power, and real‐time multimodal sensing in complex environments.

### 4.2 Machine Learning‐Based Decoupling Strategies

While structural engineering and material designs have enabled physical‐level decoupling to a certain extent, they often fall short in complex, dynamic environments where interference persists and multiple input signals cannot be easily distinguished. To address these challenges, ML‐based, data‐driven strategies have emerged as powerful tools. By extracting meaningful features from raw sensor signals and applying algorithms for classification, regression, or time‐series analysis [105, 106, 107, 108], ML approaches offer intelligent recognition and precise decoupling of multimodal physical inputs such as strain, pressure, temperature, and humidity.

**FIGURE 10:**
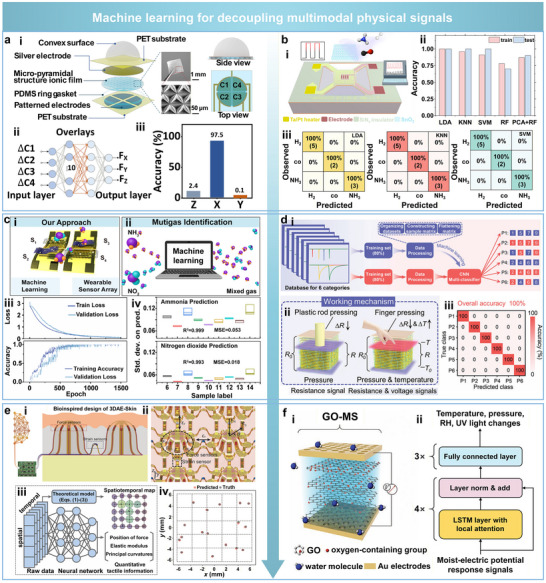
ML‐based strategies for decoupling multimodal physical signals. (a) Directional force decoupling using a backpropagation (BP) neural network. Reproduced with permission [109]. Copyright 2023, IEEE. (i) Sensor structure consisting of a domed elastomer, micropyramidal ionic membrane, and quadrupole capacitive electrodes. (ii) Architecture of the BP neural network used for directional classification. (iii) Classification accuracy of directional forces along x, y, and z directions. (b) Single‐sensor gas identification based on transient response and ML. Reproduced with permission [110]. Copyright 2025, Springer Nature. (i) Dynamic pulse‐heating strategy applied to SnO_2_ sensing layer to induce temperature‐dependent gas signatures. (ii) Comparative classification accuracy of different models. (iii) Confusion matrices indicating 100% classification accuracy of H_2_, CO, and NH_3_ using Linear Discriminant Analysis (LDA), k‐Nearest Neighbors (KNN), and Support Vector Machine (SVM). (c) ML‐enabled gas recognition in high‐humidity environments. Reproduced with permission [111]. Copyright 2024, Wiley‐VCH. (i) Hybrid architecture combining sensor array and ML model for detecting NO_2_ and NH_3_. (ii) Schematic showing signal processing pipeline. (iii) Loss and accuracy rates of the training and validation sets over 1500 epochs. (iv) Quantitative regression using partial least squares, achieving high accuracy (R^2^ = 0.999 for NO_2_, 0.993 for NH_3_). (d) Convolutional neural network (CNN)‐based multimodal classification of overlapping stimuli. Reproduced with permission [112]. Copyright 2024, Wiley‐VCH. (i) Classification framework using flattened matrix inputs into a CNN. (ii) Sensor response under plastic rod (pressure only) and finger (pressure + temperature) pressing. (iii) Confusion matrix demonstrating 100% recognition accuracy across six gesture‐related classes. (e) Decoding complex multimodal input using a deep neural network (DNN) and 3D biomimetic sensor architecture. Reproduced with permission [113]. Copyright 2024, American Association for the Advancement of Science. (i) 3DAE‐Skin configuration incorporating spatially separated sensors. (ii) Decoupled sensing of in‐plane strain and out‐of‐plane pressure. (iii) Joint prediction of contact location, elastic modulus, and curvature. (iv) 2D tactile mapping reconstructed from DNN outputs. (f) Long short‐term memory (LSTM)‐based decoupling in highly entangled multimodal environments. Reproduced with permission [114]. Copyright 2022, Wiley‐VCH. (i) Self‐powered graphene oxide single‐component multimodal sensor (GO‐MS) responsive to humidity, temperature, pressure, and light. (ii) Neural network framework integrating local attention modules with a multi‐layer LSTM architecture for accurate prediction of environmental parameters.

A relatively simple backpropagation (BP) neural network has been employed for directional decoupling of 3D forces [109]. The sensor features a domed structure with a micro‐pyramidal ionic membrane and four quadrupole electrodes (Figure 10a(i)). Directional loading generates distinctive signal distributions across the four internal capacitive channels. These features are fed into a BP neural network with hidden layers (Figure 10a(ii)), enabling accurate prediction of applied force directions. The experimental validation confirms a classification accuracy of 97.5% for x‐directional forces (Figure 10a(iii)).

In more complex scenarios such as mixed‐gas detection, where conventional structural or material‐based approaches fail to separate analytes, ML provides a robust solution [110]. A dynamic stimulation method applies pulsed thermal excitation to a SnO_2_‐based sensing layer, inducing transient response behaviors through alternating high‐ and low‐temperature cycles (Figure 10b(i)). Extracted multidimensional features are processed by algorithms such as Linear Discriminant Analysis (LDA), k‐Nearest Neighbors (KNN), and Support Vector Machine (SVM), each achieving high accuracy in both training and test sets (Figure 10b(ii)). For gases such as H_2_, CO, and NH_3_, the single‐sensor platform achieves 100% classification accuracy without requiring sensor arrays (Figure 10b(iii)), validating its strong gas decoupling performance.

In high‐humidity environments such as underground mines, the coexistence of multiple toxic gases (e.g., NH_3_ and NO_2_) can significantly increase sensing errors. To address this, a hybrid system combining a sensor array with ML has been developed for accurate gas identification (Figure 10c(i,ii)) [111]. Each sensor exhibits distinct response patterns to different gases, enabling spatial discrimination of NO_2_ and NH_3_ concentrations through a backpropagation neural network (BPNN) classifier, which achieves high recognition accuracy and low training loss over 1500 epochs (Figure 10c(iii)). Partial least squares regression is further employed for quantitative prediction, achieving excellent fitting accuracy with R^2^ values of 0.999 and 0.993 for NO_2_ and NH_3_, respectively (Figure 10c(iv)).

In human‐machine interaction scenarios, simultaneous pressure and temperature inputs often lead to overlapping sensor outputs, complicating signal decoupling. A convolutional neural network (CNN)‐based multimodal recognition strategy is implemented by constructing a multi‐input dataset and applying preprocessing techniques such as flattening and matrix transformation (Figure 10d(i)) [112]. The system accurately distinguishes different stimulus types‐ for example, plastic rod pressing (resistance change only) vs. finger pressing (combined resistance and voltage changes). The successful classification of six gesture‐based codes with 100% accuracy (Figure 10d(ii,iii)) also demonstrates the powerful feature extraction capability of CNN.

Bio‐inspired 3D architectures further facilitate spatial separation of sensing elements and improve deep feature extraction through deep neural networks (DNN) (Figure 10e(i)) [113]. In the 3DAE‐Skin, strain and force sensors are embedded at different vertical positions within dome and cage structures, enabling spatially resolved detection of normal and in‐plane forces (Figure 10e(ii)). By combining sensor array data with mechanical modeling outputs as inputs to a DNN, the system can simultaneously predict contact location, elastic modulus, and principal curvature (Figure 10e(iii)), and reconstruct a high‐resolution 2D tactile map (Figure 10e(iv)), highlighting the synergistic benefits of structural design and DNN‐based decoding.

In highly entangled sensing scenarios involving concurrent changes in temperature, humidity, pressure, and light, conventional models often fail to achieve robust decoupling. A self‐powered graphene oxide single‐component multimodal sensor (GO‐MS) is developed to tackle this challenge (Figure 10f(i)) [114]. Owing to the hygroscopic nature of graphene oxide (GO), humidity variations induce distinct electrical signals even in ambient conditions. These signals are processed using a multilayer long short‐term memory (LSTM) network with integrated local attention and fully connected layers (Figure 10f(ii)). The model achieves low predictive errors, including 0.7% for humidity, 1.3°C for temperature, 0.031 N for pressure, and 12.4 mW·cm^−^^2^ for light intensity, validating the strong decoupling performance in complex and dynamic environments.

From a deployment perspective, future research on wearable systems should focus on developing and optimizing ML frameworks that can achieve millisecond‐level inference latency and ultra‐low power consumption directly on embedded platforms. For example, an ultra‐low‐power wearable brain‐machine interface system with on‐device continual learning capabilities has demonstrated an inference latency of ≈21.5 ms and an energy consumption of 0.45 mJ per inference, yielding approximately 25 h of battery life with a 100 mAh battery [115]. Such progress highlights the feasibility of deploying adaptive, privacy‐preserving, and energy‐efficient ML models on wearable devices, which is essential for real‐time multimodal sensing and decoupling tasks in next‐generation soft electronics.

### 4.3 Machine Learning Issues and Solutions

Despite the significant promise of ML in decoupling multimodal sensing signals, which has been widely applied in stress detection, gas analysis, and environmental perception, the practical effectiveness of these approaches remains constrained due to several key challenges, particularly in data quality, model architecture, and system‐level integration. Bridging the gap between algorithmic feasibility and real‐world deployment necessitates a comprehensive understanding of the fundamental limitations currently faced by ML‐based decoupling strategies.

**FIGURE 11:**
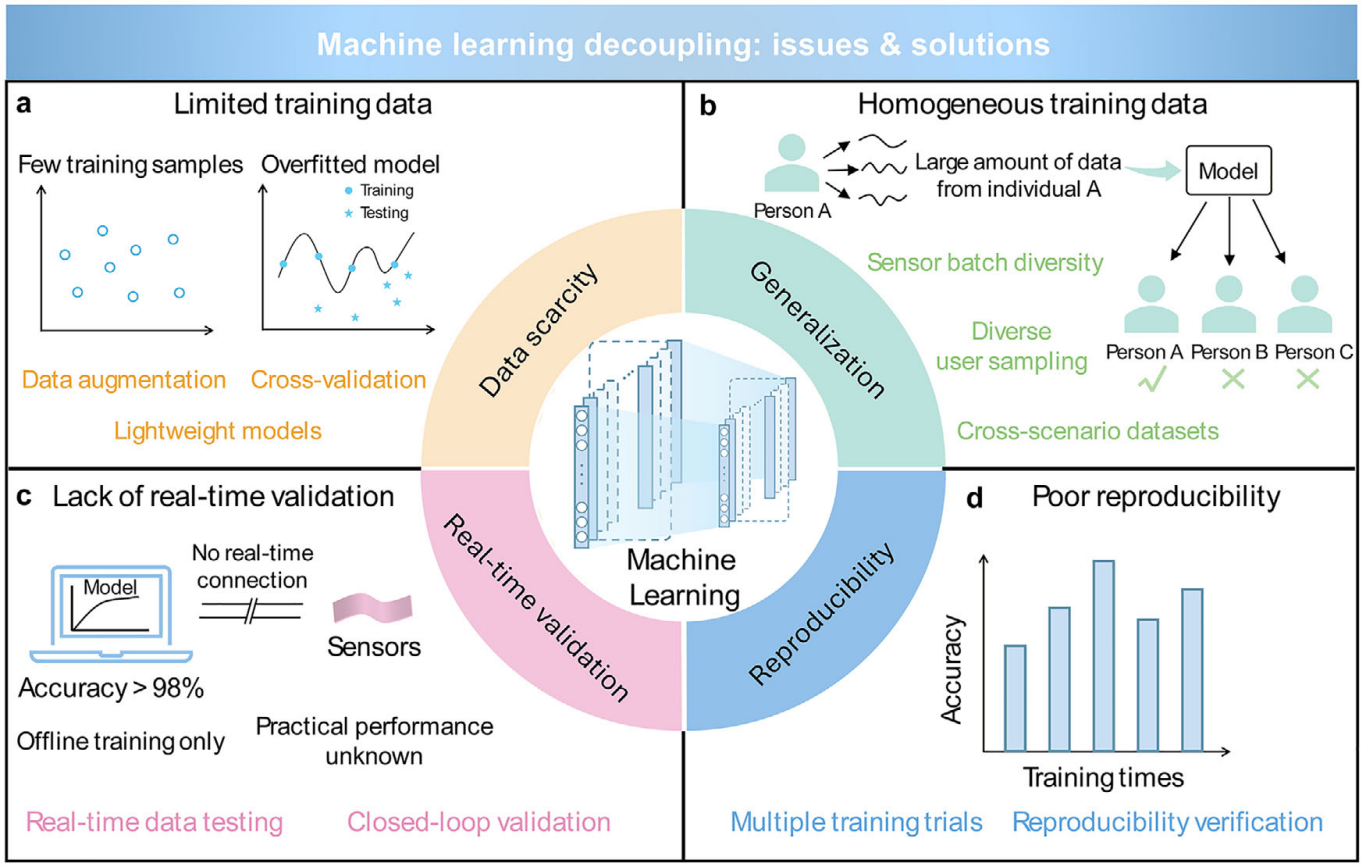
Issues and solutions for ML‐based signal decoupling. (a) Limited training data. Insufficient sample size and overly complex model architectures can lead to poor generalization. Effective mitigation strategies include data augmentation (e.g., noise injection, sliding windows), cross‐validation, and use of compact, interpretable model architectures. (b) Homogeneous training data, such as limited users, fixed postures, or constrained environmental conditions, can result in models that fail under new scenarios. Improvements involve batch‐to‐batch sensor variation, multi‐subject datasets, and expanded spatiotemporal sampling protocols. (c) Lack of real‐time validation. Many ML models are only tested offline and may perform inconsistently in dynamic real‐world conditions. Integrating real‐time sensor streams, online feedback, and closed‐loop control can improve reliability and deployment readiness. (d) Poor reproducibility. Reporting only results from a single run can mask performance instability. Recommended solutions include repeated training trials, statistical reporting (e.g., mean ± standard deviation), fixing random seeds, and standardized code structures for reproducibility verification.

First, insufficient training data remains a common bottleneck [116, 117, 118]. Many ML models are developed on limited datasets, which often results in overfitting, meaning the models perform well on training data but fail to generalize to unseen conditions (Figure 11a). While high accuracy may be reported under controlled test conditions, such models often exhibit degraded performance in dynamic or real‐world environments. To mitigate this, data augmentation strategies (e.g., noise injection, sliding windows) [119], the adoption of lightweight model architectures [120], and the use of cross‐validation [121] can improve generalization and robustness in data‐scarce scenarios.

Second, data homogeneity poses a major limitation on model generalizability (Figure 11b) [122]. For instance, wearable sensing data collected from only a few users, or environmental data restricted to specific locations or timeframes, can lead to models that fail in new contexts. Enhancing adaptability requires more diverse and representative datasets. For user‐interactive applications, data should span multiple individuals, postures, activities, and wearing styles [123]. For environmental sensing, samples should be collected across varied temporal, spatial, and device conditions to reduce overfitting and increase cross‐scenario robustness [124].

Third, many studies rely solely on offline model evaluation, neglecting real‐time validation under operational conditions (Figure 11c) [125]. While some models report high accuracy on static test sets, such metrics often fail to reflect real‐world performance. For practical deployment, trained models must be integrated into actual sensing systems and validated using live data streams. Closed‐loop validation [126], where outputs are continuously monitored and refined based on real‐time feedback, can further enhance system responsiveness and long‐term stability in dynamic environments.

Finally, reproducibility and consistency are increasingly important with the growing adoption of open‐source ML research (Figure 11d) [127]. Some studies report only peak performance from a single training session, without assessing statistical variation or repeatability. This limits reproducibility and hinders broader adoption. To address this challenge, multiple independent training trials should be conducted, with key metrics reported as mean ± standard deviation. Standardizing code structure, fixing random seeds, and implementing reproducibility validation pipelines can further reduce stochastic variance and enhance the reliability of ML‐based decoupling approaches [128].

## 5 Summary and Perspectives

This review provides a comprehensive overview of recent advances in signal decoupling strategies for soft sensors. The discussion is structured around six primary approaches: 1) stretching‐insensitive strategies, 2) bending‐insensitive strategies, 3) suppression of non‐target physical interferences (e.g., temperature, humidity, pressure, and light), 4) crosstalk‐free array‐level designs, 5) spatiotemporal multimodal decoupling strategies, and 6) ML‐assisted decoupling. Collectively, these strategies aim to enhance the accuracy, stability, and robustness of flexible sensing systems operating under complex, real‐world conditions.

1. Stretching‐insensitive strategies: To mitigate performance degradation under tensile deformation, researchers have developed diverse material and structural designs, including liquid‐solid hybrid conductors, wrinkled graphene‐elastomer composites, porous nanocomposites with dual‐mode sensing, strain‐isolated multilayer architectures, polygonal interconnects, and localized modulus reinforcement. These designs stabilize conductive pathways to provide consistent sensor output even under large tensile strain. However, challenges such as limited fabrication scalability and narrow sensing range persist. Future research should focus on scalable fabrication techniques and multifunctional structures that combine strain insensitivity with high sensitivity to the target physical signals.
2. Bending‐insensitive strategies: Bending induces asymmetric strain gradients that can disrupt signal stability. Structural strategies, including neutral mechanical plane, microstructured geometries, ultrathin conductive films, helical web designs, optimized installation angles, and off‐axis configurations, effectively minimize such interference. Despite their effectiveness, these strategies are often geometry‐dependent or limited to specific bending conditions. Moving forward, universal mechanical isolation frameworks that accommodate multidirectional and dynamic bending scenarios will be crucial.
3. Decoupling of non‐target physical interferences: Real‐world sensing environments expose devices to complex stimuli such as temperature fluctuations, humidity, pressure, and light. Decoupling strategies such as PTCR‐NTCR compensation for temperature, hydrophobic coatings to suppress moisture absorption, vertically rigid electrode arrays to reduce pressure‐induced artifacts, and photonic shielding for light immunity have demonstrated improved signal fidelity. However, most strategies are optimized for single‐parameter isolation. Future work should aim at designing integrated architectures or multifunctional materials capable of simultaneously decoupling multiple non‐target influences without compromising sensitivity or device complexity.
4. Crosstalk‐free array‐level sensing: As flexible sensors scale into high‐density arrays, inter‐channel crosstalk becomes a critical limitation. Solutions such as embedded electrode layer with common ground, isolated sensing units, micropore interlayers, serpentine interconnects, and trapezoidal prism boundary supports have been shown to suppress signal interference. However, many such systems require customized circuits or sophisticated packaging. To enable widespread adoption, efforts should prioritize simplified integration methods and modular, standardized readout protocols for scalable, low‐crosstalk array systems.
5. Multimodal decoupling strategies based on spatiotemporal designs: Simultaneous exposure to multiple stimuli, such as force, temperature, and gas, presents significant challenges for accurate sensing. Spatiotemporal decoupling strategies include stress‐directional anisotropic designs, multimodal sensing mechanisms (e.g., resistive, capacitive, and optical), selective encapsulation with conditional control, multilayer signal domain separation, magnetic vector decoding, and impedance‐based spectral analysis. These methods leverage structural anisotropy, material selectivity, and signal behavior to achieve functional decoupling. However, their complexity often impedes integration into compact and low‐cost platforms. Future research should focus on simplification of sensor architectures and development of cost‐effective backend electronics for real‐world deployment.
6. ML‐assisted decoupling: ML techniques have shown strong potential in extracting and classifying features from entangled sensor signals. However, several challenges persist, including limited data availability, inadequate generalization across users and environments, the absence of real‐time validation, and issues related to reproducibility. Addressing these challenges through the creation of diverse benchmark datasets, implementation of real‐time feedback control, and adoption of reproducibility standards will be essential for transitioning ML‐based decoupling from lab‐scale proof‐of‐concept to robust, deployable technologies.

In summary, while notable progress has been made in advanced decoupling strategies for soft sensors, further integration, standardization, and simplification are needed to translate these technologies into practical, high‐performance sensing systems for real‐world applications. For example, in addition to improving performance and integration density, future research should also address the cost and scalability of decoupling‐oriented flexible sensors. Most existing strategies rely on sophisticated materials or micro/nanofabrication processes that hinder large‐scale production. Incorporating low‐cost materials, printable electrodes [129, 130], and roll‐to‐roll manufacturing techniques could greatly enhance the translational potential of these systems for practical applications [131, 132].

Looking ahead, the next stage of single‐modal and multimodal sensing development will depend on the convergence of mechanical design, electronic integration, and intelligent data processing. Establishing unified decoupling frameworks that seamlessly couple structural, electrical, and algorithmic levels could enable real‐time, energy‐efficient, and context‐aware sensing. From the front‐end perspective, co‐designing sensors and readout circuits to minimize device footprint and enhance integration density will be essential for achieving compact, low‐power systems. From the back‐end perspective, incorporating advanced deep learning algorithms together with cloud‐based data processing can further reduce the computational burden of on‐device analysis, thereby enabling smaller and lighter front‐end units without sacrificing recognition accuracy. Moreover, expanding the database of multimodal signal patterns and integrating self‐learning or adaptive ML models will be crucial to bridge the gap between laboratory demonstrations and practical, autonomous systems for wearable healthcare, human‐machine interfaces, and soft robotics.

## Conflicts of Interest

The authors declare no conflict of interest.

## Data Availability

The authors have nothing to report.
